# Epigenetic regulators polyphenols in neurodegenerative diseases: a promising intervention strategy

**DOI:** 10.1080/07853890.2026.2634566

**Published:** 2026-03-31

**Authors:** Lu-Hao Li, Yi Huang, Xiao-xiang Wang, Cheng-cheng Xu, Lei Wu, Ke-lin He, De-xiong Han, Zhuo Chang, Jia-qi Wang, Rui-jie Ma

**Affiliations:** a The Third School of Clinical Medicine (School of Rehabilitation Medicine), Zhejiang Chinese Medical University, Hangzhou, China; b Key Laboratory of Acupuncture and Neurology of Zhejiang Province, The Third School of Clinical Medicine (School of Rehabilitation Medicine), Zhejiang Chinese Medical University, Hangzhou, China; c Department of Acupuncture, The Third Affiliated Hospital of Zhejiang Chinese Medical University (Zhongshan Hospital of Zhejiang Province), Hangzhou, China; d School of Basic Medical Sciences, Heilongjiang University of Chinese Medicine, Harbin, China

**Keywords:** Polyphenols, epigenetic modulation, neurodegeneration, curcumin, resveratrol, epigallocatechin-3-gallate, histone acetylation, miRNA regulation

## Abstract

**Background:**

Neurodegenerative diseases are complex disorders characterized by the progressive loss of neuronal structure and function, involving pathological mechanisms such as oxidative stress, chronic inflammation, protein misfolding, and impaired synaptic plasticity. Recent studies have revealed that epigenetic regulation plays a critical role in the onset and progression of these diseases, including mechanisms such as DNA methylation, histone modifications, and non-coding RNA regulation. Natural polyphenolic compounds, known for their safety and multi-target properties, have emerged as promising candidates for neuroprotection and therapeutic intervention.

**Methods:**

This review summarizes recent advances in the neuroprotective effects of polyphenols in neurodegenerative diseases through epigenetic mechanisms. It focuses on their regulation of DNA methyltransferase activity, histone acetylation status, and non-coding RNA expression, as well as their influence on neurotrophic factors, inflammatory mediators, and synapse-related gene expression.

**Results:**

Polyphenolic compounds such as curcumin, resveratrol, and epigallocatechin gallate (EGCG) have been shown to modulate epigenetic enzyme activity, alleviate neuroinflammation, improve mitochondrial function, and promote neuroregeneration, demonstrating multi-level neuroprotective effects. However, clinical translation is limited by low oral bioavailability, rapid metabolism, and insufficient brain-targeting capacity. Emerging strategies including nano-delivery systems, prodrug design, and omics technologies hold promise in overcoming these limitations and enhancing therapeutic efficacy.

**Conclusions:**

Polyphenols exert multi-target regulatory effects *via* epigenetic mechanisms in neurodegenerative diseases, showing great potential for intervention. In the future, integrating precision nutrition, personalized treatment, and multi-omics approaches may facilitate the development of novel epigenetic-based strategies for the prevention and treatment of neurodegenerative disorders.

## 1. Introduction

Neurodegenerative diseases constitute a class of intricate conditions marked by the gradual deterioration of neuronal structure and function, often associated with sustained inflammation, oxidative damage, aberrant protein folding, and disruptions in neuroplasticity [1–4]. Despite the incomplete understanding of their pathogenic basis, growing research suggests that epigenetic mechanisms are fundamentally involved in initiating and driving the development of these disorders [5–7]. Epigenetic mechanisms such as DNA methylation, histone modification, and non-coding RNA (ncRNA) regulation profoundly influence neuronal survival and functional alterations under conditions of stress, inflammation, and metabolic imbalance by modulating gene expression patterns [8,9]. These reversible modifications constitute a regulatory network that not only underlies the nervous system’s adaptability to external stimuli but also offers novel insights and potential strategies for the prevention and treatment of neurodegenerative diseases [5].

In this context, natural polyphenols are increasingly recognized for their potential to modulate neurodegenerative processes, owing to their broad accessibility, favorable safety, and ability to act on multiple molecular targets [10]. Unlike conventional drugs that typically act on a single pathological pathway, polyphenols exhibit neuroprotective potential in the central nervous system (CNS) through synergistic modulation of multiple signaling cascades [11]. They not only directly scavenge free radicals and suppress inflammatory responses but also participate deeply in epigenetic regulation, thereby reshaping the expression patterns of key genes [11]. Research indicates that polyphenols are capable of suppressing DNA methyltransferase (DNMT) activity, regulating histone acetylation dynamics, as well as enhancing the expression of neurotrophic factors, anti-inflammatory agents, and synaptic plasticity-related genes [11,12]. At the same time, they suppress the excessive activation of pro-inflammatory and pro-apoptotic pathways, thereby playing an essential role in maintaining neural homeostasis and regulating cell fate [11].

More importantly, the neuroprotective effects mediated by these epigenetic mechanisms are systemic rather than confined to a single molecular pathway [13]. Owing to this property, polyphenols may function as prophylactic agents during the initial phases of prevalent conditions like Alzheimer’s (AD) and Parkinson’s disease (PD), while also acting as modulators that can decelerate disease progression and enhance neural performance [14,15].

Therefore, thoroughly exploring the epigenetic regulation exerted by polyphenolic compounds on immune activity, neuronal processes, and cellular outcomes within the CNS can deepen insights into their neuroprotective roles while simultaneously opening novel avenues for both nutritional strategies and precision medicine [16,17]. As related studies continue to advance, the epigenetic regulatory properties of polyphenols are expected to become increasingly elucidated, potentially paving the way for novel multi-target strategies in the prevention and treatment of neurodegenerative diseases [18].

## 2. Materials and methods

### 2.1. Literature search strategy

This review aims to systematically summarize the neuroprotective effects and molecular mechanisms of natural polyphenolic compounds in neurodegenerative diseases through epigenetic regulatory pathways. A comprehensive literature search was conducted across several major international academic databases, including PubMed, Web of Science, Embase, and Google Scholar. The search was limited to publications released prior to November 2025, to ensure the timeliness and completeness of the included studies.

A combination of subject headings and free-text terms was employed, utilizing Boolean operators (AND, OR) to construct search strategies. Core search terms included but were not limited to: ‘polyphenols’, ‘epigenetic regulation’, ‘DNA methylation’, ‘histone modification’, ‘non-coding RNA’, ‘neurodegenerative diseases’, ‘Alzheimer’s disease’, ‘Parkinson’s disease’, ‘curcumin’, ‘resveratrol’, and ‘epigallocatechin-3-gallate (EGCG)’. Synonyms, abbreviations, and related derivatives of these terms were also included to maximize coverage of relevant literature.

In addition, manual screening of the reference lists of selected articles was performed to identify potentially relevant studies that may have been missed during the initial database searches.

### 2.2. Inclusion and exclusion criteria

Studies included in this review were required to meet the following criteria: they had to be peer-reviewed articles published in English, focusing on natural polyphenolic compounds such as curcumin, resveratrol, and epigallocatechin-3-gallate (EGCG). The studies must explicitly investigate epigenetic regulatory mechanisms, including DNA methylation, histone modification, or non-coding RNA regulation, and be related to neurodegenerative diseases such as Alzheimer’s disease, Parkinson’s disease, amyotrophic lateral sclerosis, multiple sclerosis, or pathological processes of the central nervous system.

Studies were excluded if they were not published in English, were unrelated to the nervous system or neurodegenerative diseases, did not address epigenetic mechanisms or only provided general descriptions of antioxidant or anti-inflammatory effects, or were published as conference abstracts, proceedings, editorials, opinion articles, study protocols, or letters. Additionally, studies lacking clear experimental evidence or sufficient methodological details, as well as duplicate publications or those with substantially overlapping content, were also excluded.

### 2.3. Literature coverage

The literature included in this review primarily covers research from the past two decades (approximately 2005–2025), with a focus on recent advances in epigenetics, neuroinflammation, neuroprotection, and precision nutritional interventions. Through systematic screening and integrated analysis, this review aims to outline the epigenetic regulatory features of natural polyphenols in various neurodegenerative disease models, elucidate their multi-target neuroprotective mechanisms, and provide a theoretical foundation for future individualized nutritional interventions and precision therapeutic strategies.

## 3. Epigenetic mechanisms in the nervous system

### 3.1. DNA methylation

DNA methylationA, a key reversible process involved in epigenetic gene regulation, typically occurs within CpG islands located in promoter regions, which are DNA segments rich in cytosine and guanine dinucleotides [19]. Through the enzymatic action of DNMTs, a methyl group derived from S-adenosylmethionine (SAM) is attached to the fifth carbon of the cytosine ring, which leads to the production of 5-methylcytosine (5-mC) [20]. This modification can recruit methyl-CpG-binding proteins, such as methyl-CpG-binding protein 2 (MeCP2), along with associated repressive transcriptional complexes, thereby hindering the binding of transcription factors and repressing target gene expression [21]. DNA methylation is primarily carried out by DNMT1, DNMT3A, and DNMT3B, whereas its active removal involves enzymes from the TET protein family (TET1, TET2, and TET3), which progressively oxidize 5-mC into intermediate derivatives such as 5-hydroxymethylcytosine (5-hmC) [22,23]. The functional roles of methylation modifications also rely on a series of reader proteins that recognize 5-mC and 5-hmC, thereby influencing chromatin structure and gene expression states [24].

In the CNS, DNA methylation plays a crucial role in the proliferation and differentiation of neural stem cells (NSCs), as well as in neuronal fate determination, synapse formation, and the regulation of synaptic plasticity [25,26]. The demethylation process is equally important, contributing significantly to learning and memory formation, the maintenance of neural plasticity, and tissue repair following injury [27]. When this dynamic regulatory mechanism becomes imbalanced, particularly through abnormal methylation occurring in the promoter regions of key neurofunctional genes, it can lead to gene silencing or inactivation, disrupt the excitatory and inhibitory balance of neurons, and cause abnormal neural electrical activity [28]. Such modifications are significantly correlated with a range of neurological and psychiatric illnesses, among them AD, PD, MDD, and epilepsy [29–32].

Taking epilepsy as an example, the gene encoding the delta subunit of the gamma-aminobutyric acid type A receptor (GABAA receptor), GABRD, plays a key role in maintaining extrasynaptic inhibitory tone [33]. Downregulation of its expression enhances neuronal excitability and induces synchronized discharges [33]. In animal models of status epilepticus (SE), the CpG island within the promoter region of GABRD exhibits significant hypermethylation, primarily mediated by DNA methyltransferase 1 (DNMT1) [34]. DNMT1 expression inhibits GABRD transcription; however, targeted intervention can restore gene activity, suggesting that this methylation-based mechanism is critically involved in the development of epilepsy [34].

**Figure 1.**
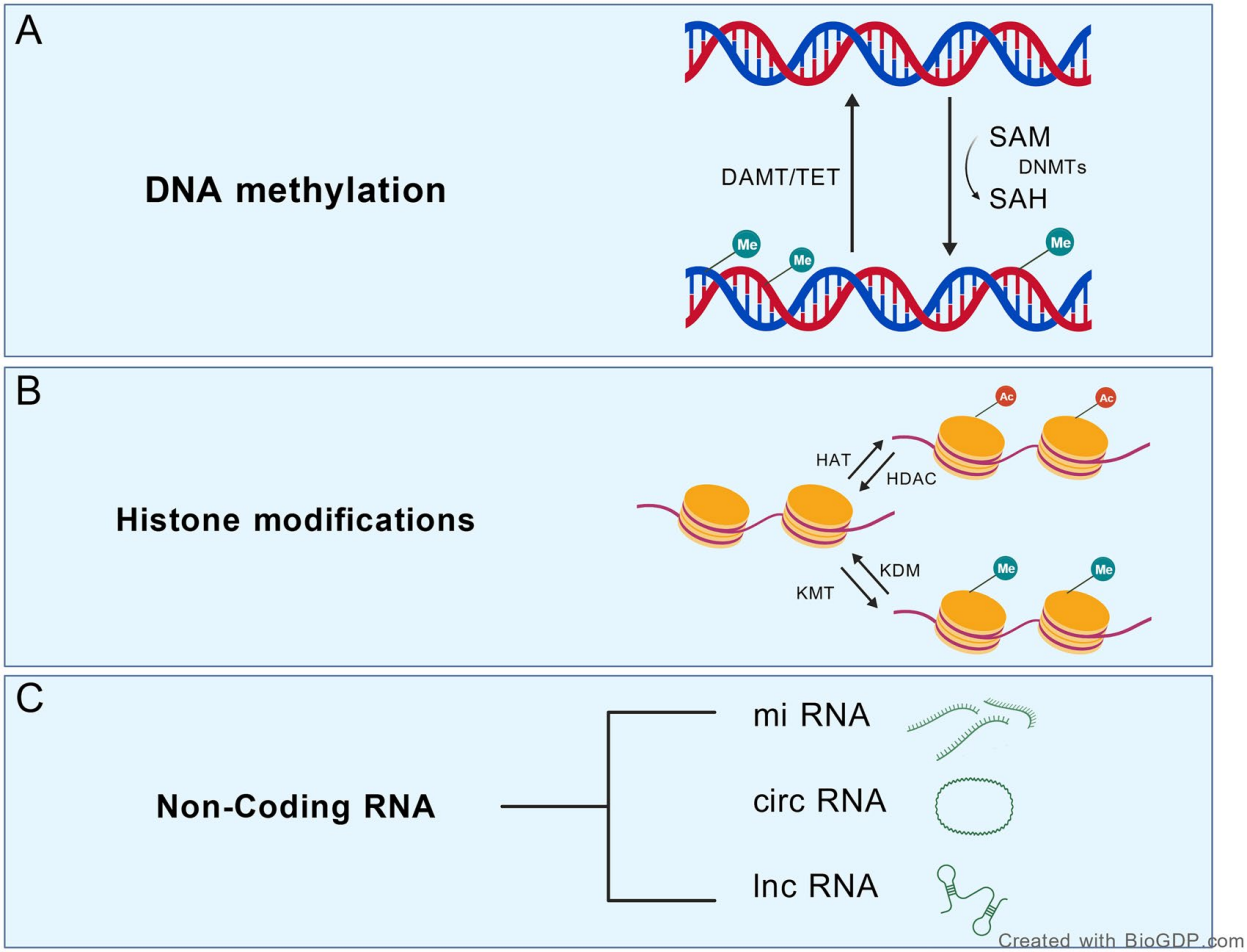
Overview of epigenetic regulatory mechanisms: (A) DNA methylation and demethylation are catalyzed by DNA methyltransferases (DNMTs) and Ten-eleven translocation enzymes (TETs), which add or remove methyl groups at CpG sites to modulate gene activation or silencing. This process uses S-adenosylmethionine (SAM) as a methyl donor and produces S-adenosylhomocysteine (SAH) as a byproduct; certain methylation reactions also involve methylated nucleotide methyltransferases (MNMTs). (B) Histone modifications, including acetylation (Ac)/deacetylation and methylation (Me)/demethylation, are mediated by HATs, HDACs, lysine methyltransferases (KMTs), and lysine demethylases (KDMs). These modifications alter chromatin accessibility, thereby promoting or repressing transcription. (C) Non-coding RNA (ncRNA)-mediated regulation, such as by miRNAs, lncRNAs, and circRNAs, fine-tunes gene expression by modulating mRNA stability and translation efficiency. These mechanisms are widely conserved across organisms and act in concert to maintain the dynamic balance and precise control of gene expression. Created with BioGDP.com [108]. DNMTs: DNA methyltransferases; TETs: Ten-eleven translocation enzymes; SAM: S-adenosylmethionine; SAH: S-adenosylhomocysteine; HAT: histone acetyltransferase; KMT: lysine methyltransferase; HDAC: histone deacetylase; KDM: lysine demethylase; Ac: acetylation; Me: methylation; miRNA: microRNA; circRNA: circular RNA; lncRNA: long non-coding RNA.

In summary, DNA methylation, as a reversible epigenetic modification, plays a crucial role in neural development, functional maintenance, and disease pathogenesis. Disruption of its regulation not only alters the balance between neuronal excitation and inhibition, but also presents opportunities for therapeutic intervention aimed at preventing and managing neurological diseases. Figure 1 illustrates the major epigenetic regulatory mechanisms involved in the nervous system, including DNA methylation, histone modifications, and regulation mediated by non-coding RNAs, which collectively influence gene expression and neuronal function.

### 3.2. Histone modifications in the nervous system

Histone modifications constitute a fundamental layer of epigenetic regulation, influencing gene transcription and being deeply involved in neural development, maintenance of neuronal function, response to injury, and the progression of neurological disorders [27,35]. This mechanism relies on post-translational modifications occurring at specific sites on the N-terminal tails of histones, which alter chromatin conformation and influence transcription factor binding as well as RNA polymerase activity, thereby achieving precise control of gene expression [36].

Due to the structural complexity, cellular diversity, and delicate developmental processes of the nervous system, gene regulation is highly sensitive to external and internal cues. Histone modifications serve as a crucial link between external signals and gene regulation within this context [37].

During neural development, NSCs must maintain a balance between self-renewal and differentiation [38]. Histone acetylation and methylation are the predominant forms of modification in this process. Acetylation, catalyzed by histone acetyltransferases (HATs) such as CBP and p300, loosens chromatin structure and enhances transcriptional activity [39]. During the differentiation of NSCs into neurons, promoters of differentiation-related genes such as Neurog2 are typically enriched with H3K9ac [40]. Correspondingly, histone deacetylases (HDACs) such as HDAC1 and HDAC2 maintain chromatin compaction by removing acetyl groups, thereby preventing aberrant gene activation and preserving cell phenotype stability in both NSCs and mature neurons [41]. HDAC3 suppresses the expression of regeneration-associated genes in injury conditions, thereby limiting axonal regeneration, while HDAC6 influences axonal transport by regulating microtubule acetylation status [42]. SIRT1, an NAD^+^-dependent HDAC, links metabolic state to epigenetic regulation and plays a critical role in modulating NSC fate and stress responses [43].

Histone methylation exhibits greater functional complexity than acetylation, with its regulatory effects depending on both the specific residue modified and the degree of methylation [44]. H3K4me3 is typically associated with transcriptional activation and is frequently enriched at the promoter regions of genes involved in neural development [45]. In contrast, H3K27me3 and H3K9me3 are generally linked to transcriptional repression, maintaining the silenced state of genes that are not required for activation [45]. In NSCs, many key developmental genes carry both H3K4me3 and H3K27me3 marks, representing a poised state that allows rapid activation when needed [46]. The demethylase Jmjd3 (also known as KDM6B) facilitates the removal of H3K27me3, thereby rapidly initiating the expression of neuron-specific genes [46]. In addition, H3K9me3 mediated by SETDB1 precisely restricts the spatial distribution of neural progenitor cells, contributing to the formation of the layered structure of the cerebral cortex [41].

Recent advances have progressively revealed the functional significance of newly identified histone modifications within the nervous system [47]. Crotonylation, such as H3K9cr, is catalyzed by p300 and enhances the expression of neuron-specific genes [48]. Lactylation, such as H4K12la, is regulated by metabolic status; under hypoxic or inflammatory conditions, lactate promotes the activation of inflammatory genes in microglia [49,50]. Serotonylation involves the attachment of serotonin to the H3Q5 residue through transglutaminase 2 (TGM2), which enhances H3K4me3-mediated transcription and promotes neuronal differentiation [51]. These novel modifications connect metabolic activity, inflammatory signaling, and transcriptional control, thus providing deeper insights into how epigenetic mechanisms operate within neural tissues.

In neurodegenerative diseases, histone modifications frequently exhibit widespread abnormalities [40]. In AD, the marked upregulation of HDAC2 in the hippocampus leads to decreased acetylation levels of histones H3 and H4, thereby suppressing the expression of key synaptic plasticity regulators, such as BDNF, and ultimately resulting in impaired memory formation and learning capacity [52]. In PD models, alpha-synuclein (α-syn) induces the upregulation of EHMT1 and EHMT2 and increases H3K9me2 levels, which in turn represses the transcription of genes involved in synaptic vesicle transport, such as SNAP25, Synapsin1, vGLUT1, and PSD95[53]. In schizophrenia (SZ), mutations in SETD1A affect enhancer activity, while GABAergic neuron-related genes such as GAD1 are downregulated due to increased H3K27me3, resulting in neuronal dysfunction [41]. In major depressive disorder (MDD), differential acetylation patterns are observed across distinct brain regions, reflecting region-specific epigenetic abnormalities [54].

During neural injury and regeneration, histone modification states are considered key determinants of regenerative capacity [55]. Following peripheral nervous system (PNS) injury, HATs are upregulated and enhance acetylation levels, thereby activating the expression of regeneration-associated genes (RAGs) and promoting axonal regrowth [42]. In contrast, within the CNS, HDAC3 and bromodomain-containing protein 4 (BRD4) maintain chromatin in a repressive state, hindering the initiation of regeneration, whereas inhibition of these factors can enhance axonal regeneration efficiency [42]. Microtubule acetylation exhibits similarly distinct effects: in the PNS, HDAC5-mediated deacetylation increases microtubule dynamics, facilitating growth cone advancement, while in the CNS, inhibition of HDAC6 enhances microtubule stability and supports axonal elongation [56,57]. In addition, HDACs and bromodomain and extraterminal domain (BET) proteins within glial cells modulate the inflammatory response and neurotrophic factor expression, thereby indirectly shaping the neural repair environment [58,59].

In summary, histone modifications regulate the precision and plasticity of gene expression by altering chromatin structure, playing a central role in neural development, functional maintenance, and regeneration within the nervous system.

#### 3.2.1. Histone methylation machinery and reader proteins

Unlike histone acetylation, which generally promotes gene transcription activation, the biological effects of histone methylation depend on the specific amino acid residues being modified, the degree of methylation (i.e. mono-, di-, or tri-methylation), and the functional proteins subsequently recruited [60]. Overall, this regulatory process is coordinated by three major classes of factors: histone methyltransferases (‘writers’), demethylases (‘erasers’), and methylation-binding proteins (‘readers’) [60].

In the methylation ‘writing’ process, lysine methyltransferases (KMTs), such as the SETD1/MLL family, EZH2, EHMT1/2, and SETDB1, catalyze methylation at key sites like H3K4, H3K9, and H3K27 [61–63]. In the nervous system, these enzymes help establish finely tuned transcriptional regulation, contributing to neuronal fate determination, activity-dependent gene expression, and long-term memory formation [64]. In neurodegenerative diseases such as Alzheimer’s and Parkinson’s, these methylation patterns are often disrupted early, leading to suppressed expression of neurotrophic and synapse-related genes, and accelerating cognitive decline and neuronal damage [53,65].

Histone demethylases (KDMs), which mediate the ‘erasure’ of methylation marks, are equally essential. Members of the KDM5, KDM6 (JMJD3/UTX), and KDM4 families can dynamically remove methyl marks in response to neuronal activity, metabolic shifts, or inflammatory signals, thereby enhancing chromatin plasticity and transcriptional adaptability [66]. In the context of neurodegeneration, impaired demethylation can leave chromatin in a persistently repressive state, limiting the neuronal response to stress and injury [67].

In addition to the processes of methylation writing and erasing, the functional outcome of histone methylation also relies heavily on specific recognition mechanisms [68]. Methylation reader proteins are able to detect methylated histone tails and translate these modifications into defined transcriptional responses or changes in chromatin organization [68]. For example, heterochromatin protein 1 binds to H3K9me3 and promotes heterochromatin formation, while Polycomb group complexes associate with H3K27me3 to maintain long term gene repression [68,69]. When the function or localization of these reader proteins is disrupted, proper transcriptional regulation may fail to occur even in the presence of methylation marks, ultimately leading to synaptic dysfunction and neurodegenerative alterations [69].

Overall, histone methylation should not be viewed as a static epigenetic modification but rather as a dynamic and highly coordinated regulatory system. Disruption at any level, including methylation deposition, removal, or recognition, can interfere with normal gene expression programs in neurons and constitutes an important molecular basis for the onset and progression of neurodegenerative diseases.

#### 3.2.2. ATP-dependent chromatin remodeling complexes

In addition to covalent modifications of histones, the three-dimensional organization of chromatin and the accessibility of DNA are also tightly regulated by ATP-dependent chromatin remodeling complexes [70]. These multi-protein assemblies utilize the energy from ATP hydrolysis to drive nucleosome sliding, repositioning, or eviction along the DNA, thereby dynamically modulating the access of transcription factors and the transcriptional machinery to target genes [70]. In the nervous system, neuronal responses to synaptic plasticity, learning and memory processes, as well as environmental stimuli, rely on highly plastic and rapidly reversible transcriptional responses, making this regulatory mechanism particularly crucial [71].

The major families of chromatin remodeling complexes identified to date include SWI/SNF (referred to as BAF complexes in mammals), ISWI, CHD, and INO80. Among them, the SWI/SNF or BAF complex plays a central role in neurodevelopment and the maintenance of mature neuronal functions. During neuronal differentiation, the subunit composition of the BAF complex undergoes a specific switch to form neuron-specific nBAF complexes, which are essential for dendritic development, synapse formation, and memory consolidation [72]. Numerous studies have shown that alterations in the composition or function of the BAF complex are closely associated with impaired synaptic plasticity and neurodegenerative processes [73,74].

CHD family proteins regulate nucleosome spacing and enhancer accessibility, contributing to activity-dependent transcriptional regulation and neuronal survival. When CHD function is compromised, gene expression programs may become dysregulated, leading to cognitive deficits and various neurological disorders [75]. ISWI complexes are mainly involved in maintaining higher-order chromatin structure, ensuring stable expression of neuronal identity genes while preventing aberrant activation of non-specific gene expression [76].

An increasing body of evidence suggests that dysfunction in chromatin remodeling is an important, yet long-underestimated, pathogenic mechanism in neurodegenerative diseases [77]. As aging or disease progresses, the efficiency of chromatin remodeling tends to decline, reducing the ability of neurons to flexibly adjust gene expression in response to metabolic stress, inflammation, or toxic protein aggregates [77]. Therefore, ATP-dependent chromatin remodeling represents a key epigenetic layer that links environmental signals, transcriptional regulation, and neuronal vulnerability [78].

### 3.3. Non-coding RNA regulation

In the CNS, non-coding RNAs (ncRNAs) participate in key processes such as neural development, synaptic plasticity, neural injury repair, and neurodegenerative diseases through intricate and finely tuned molecular mechanisms [79]. ncRNAs are mainly categorized into microRNAs (miRNAs), long non-coding RNAs (lncRNAs), and circular RNAs (circRNAs), all of which contribute to neural regulation through diverse mechanisms [80].

#### 3.3.1. MicroRNAs (miRNAs)

Regarding microRNAs (miRNAs), these small RNA molecules primarily exert negative regulation of gene expression by binding specifically to the 3′ untranslated regions (3′-UTRs) of target messenger RNAs (mRNAs), leading to mRNA degradation or translational repression [81]. In the nervous system, miRNAs are highly expressed, undergoing stepwise processing by Drosha and Dicer before becoming functionally active within the RNA-induced silencing complex (RISC) complex, where they fine-tune the expression of genes critical for neuronal activity [82].

During early neural development, miRNAs control the differentiation fate of neural progenitor cells [83]. In mature neurons, they regulate synaptic structure and gene expression to maintain functional homeostasis and feedback control [83]. For instance, miR-124 is highly expressed in neurons and maintains neuronal phenotype stability by repressing non-neuronal genes while also modulating chromatin-remodeling factors, thereby linking post-transcriptional regulation with epigenetic control [84,85].

In neural injury and regeneration, miRNAs finely modulate cell fate by regulating signaling pathways involved in apoptosis, autophagy, inflammation, and axonal regeneration [86]. Some miRNAs promote axonal regrowth by inhibiting phosphatase and tensin homolog (PTEN) and activating the mammalian target of rapamycin (mTOR) pathway, while others influence neuronal survival or death under stress conditions such as ischemia or trauma by targeting apoptotic regulators including the Caspase family and B-cell lymphoma 2 (Bcl-2)[87].

#### 3.3.2. Long non-coding RNAs (lncRNAs)

At the level of long non-coding RNAs (lncRNAs), these RNA molecules, typically longer than 200 nucleotides, generally do not encode proteins but regulate gene expression through interactions with DNA, RNA, or proteins [88]. They participate in chromatin modification, transcriptional regulation, and post-transcriptional processes such as RNA splicing, stability, and translation, thereby playing crucial roles in multilayered regulatory networks within the nervous system [89]. lncRNAs display high tissue specificity and finely tuned expression patterns in the nervous system, enabling cross-cell-type communication and regulation among neurons, glial cells, neural stem cells, and immune cells [89].

Functionally, lncRNAs can act as competing endogenous RNAs (ceRNAs) that sponge miRNAs, thereby relieving repression of neuronal mRNA targets, or they can directly participate in pathological processes [90]. For example, lncRNAs such as MEG3, NEAT1, and H19 are commonly upregulated in various neurodegenerative diseases and contribute to disease pathology by regulating tau protein phosphorylation, amyloid beta (Aβ) production, mitochondrial autophagy, inflammatory cytokine release, and microglial activation [91–94]. In addition, lncRNAs can modulate the expression of N6-methyladenosine (m6A) modification enzymes or serve as modification substrates themselves, affecting their stability and functional state, thus linking epigenetic regulation with RNA metabolism [95,96]. In central nervous system disorders such as ischemia-reperfusion injury following stroke, lncRNAs influence angiogenesis and autophagy, exerting significant effects on functional recovery and disease progression, and demonstrating considerable therapeutic potential [97,98].

#### 3.3.3. Circular RNAs (circRNAs)

Circular RNAs (circRNAs) are a class of covalently closed-loop RNA molecules formed through back-splicing of precursor mRNAs [99]. Due to the absence of a 5′ cap and a 3′ tail, they exhibit high stability and are abundantly expressed in the nervous system, displaying distinct developmental stage dependent and cell type specific expression patterns [100]. The primary mechanism of circRNA function involves acting as molecular sponges for miRNAs, competitively binding to them and thereby indirectly regulating the expression of target mRNAs [101]. Through this mechanism, circRNAs participate in processes such as synaptic plasticity, axon guidance, neuroinflammation, and cell survival [102,103].

In addition, some circRNAs interact with RNA-binding proteins to modulate mRNA transport, splicing, or local translation [104]. Under pathological conditions such as CNS injury, neurodegenerative diseases, and chronic neuropathic pain, circRNA levels undergo significant alterations. Functioning as ceRNAs that interact with miRNAs, they modulate the transcriptional activity of genes connected to inflammatory processes, oxidative stress responses, ion channel performance, and synaptic plasticity [105,106]. Furthermore, circRNAs can interact with both miRNAs and lncRNAs, collaboratively contributing to the dynamic regulation of the CNS and playing important roles in disease progression [106,107].

In summary, miRNAs, lncRNAs, and circRNAs not only exert their respective regulatory functions within the nervous system but also interact with one another to produce synergistic effects.

### 3.4. Epigenetic regulation and risk factors in neurodegenerative diseases

While the aforementioned sections have summarized the fundamental mechanisms of epigenetic regulation in the nervous system, increasing evidence suggests that these mechanisms are highly sensitive to environmental and lifestyle influences. Understanding how external risk factors interact with epigenetic regulation is essential for elucidating the multifactorial etiology of neurodegenerative diseases.

#### 3.4.1. Epigenetic impacts of lifestyle and environmental factors

Neurodegenerative disorders arise from the prolonged interplay among multiple contributing factors [109]. The core mechanisms involve not only cellular pathological changes such as protein misfolding, mitochondrial dysfunction, disruption of the blood–brain barrier (BBB), and neuroinflammation, but are also closely related to epigenetic regulation [5,109,110]. In recent years, numerous studies have demonstrated that lifestyle and environmental factors, including nutritional metal imbalance, exposure to environmental toxins, smoking, and coffee consumption, as well as the aging process itself, may induce persistent transcriptional abnormalities in the nervous system by influencing epigenetic mechanisms such as DNA methylation, histone modification, and non-coding RNA (ncRNA) expression [111,112]. These changes collectively contribute to neuronal dysfunction and degenerative alterations.

Epidemiological evidence indicates that smoking and coffee consumption are closely associated with a reduced risk of PD [113,114]. However, the underlying mechanisms extend beyond a single neuroprotective effect and involve complex epigenetic regulatory processes [115,116]. Caffeine, as an adenosine A2A receptor antagonist, not only alleviates glutamate excitotoxicity at the synaptic level but also promotes the clearance of α-synuclein by enhancing autophagy and activating the AMP-activated protein kinase (AMPK) and sirtuin 3 (SIRT3) signaling pathways [116]. In addition, caffeine and the coffee component eicosanoyl-5-hydroxytryptamide (EHT) have been found to synergistically increase the methylation level of protein phosphatase 2 A (PP2A), thereby reducing the accumulation of phosphorylated pathogenic proteins [113]. This process involves the regulation of protein phosphatase methylesterase-1 (PME-1) activity and exemplifies a typical enzyme-mediated epigenetic modification pathway [113].

In contrast, the neuromodulatory mechanisms activated by nicotine in tobacco smoke may exert early-stage intervention in Parkinson’s disease pathology by inhibiting dopamine transporter (DAT) function and increasing synaptic dopamine concentrations [114]. These neurotransmitter alterations are often accompanied by long-term adjustments in histone acetylation and non-coding RNA (ncRNA) expression, indicating a close relationship between neuronal activity and chromatin plasticity [117].

In addition to neurotransmitters and metals, environmental toxins, particularly neurotoxic pesticides such as paraquat and dieldrin, heavy metals including lead, arsenic, and aluminum, as well as certain organic solvents, are also important contributors to neurodegenerative diseases [117,118]. These toxic substances can impair mitochondrial complex function, leading to ATP depletion and excessive production of ROS [119]. When acting together with dopamine and metal ions, they can trigger Fenton and Haber reactions that result in oxidative damage to DNA, proteins, and lipids [118].

Oxidation of guanine at CpG sites, producing 8-oxo-2′-deoxyguanosine (8-oxodG), inhibits methylation of neighboring cytosine residues, which may lead to site-specific hypomethylation. At the same time, abnormal promoter methylation has been observed in several disease-related genes, such as amyloid precursor protein (APP), tau protein (Tau), glycogen synthase kinase 3 beta (GSK3β), and alpha-synuclein (SNCA), with SNCA hypomethylation being a representative example [118].

It is noteworthy that early exposure to these toxicants may induce persistent epigenetic alterations during critical developmental stages, which can later be reactivated in adulthood or aging. This phenomenon may help explain why some individuals develop neurodegenerative pathology following the same toxic exposure, whereas others remain unaffected, emphasizing the individual variability of epigenetic regulation in mediating the conversion of environmental exposure into disease risk [119,120].

#### 3.4.2. Aging and individual epigenetic susceptibility

In addition, aging itself is one of the most significant risk factors for neurodegenerative diseases, and one of its core features is the decline in epigenetic stability [117]. As the aging process progresses, global DNA methylation levels tend to decrease, leading to the gradual opening of genomic regions that were previously transcriptionally silent. At the same time, certain actively transcribed regions may become repressed due to reduced histone acetylation and enhanced heterochromatin formation [117,121].

In summary, various risk factors for neurodegenerative diseases, including nutritional metal imbalance, environmental toxin exposure, lifestyle differences, and natural aging, can induce epigenetic abnormalities that alter neuronal transcriptional activity, thereby disrupting structural stability and functional integrity [119]. Together, these processes emphasize the central regulatory role of epigenetic mechanisms in maintaining neural homeostasis and mediating neurodegenerative changes, providing an important theoretical basis and potential targets for early prediction and intervention.

### 3.5. Enhancer dynamics and three-dimensional chromatin architecture

In recent years, epigenomic studies have revealed that epigenetic regulation in the nervous system is not limited to chemical modifications along linear DNA. It also involves dynamic changes in enhancer activity and the remodeling of three-dimensional chromatin architecture. Neurons exhibit highly cell-type-specific and activity-dependent enhancer landscapes [122]. These enhancers are typically enriched with hallmark histone modifications such as H3K4me1 and H3K27ac and play essential roles in regulating the expression of genes associated with synaptic function, neuronal plasticity, and stress responses [122].

Enhancers exert their regulatory effects by establishing spatial contacts with their target gene promoters through chromatin loops, thereby enabling long-range transcriptional control [123]. This process primarily depends on the coordinated actions of chromatin architectural proteins, such as CCCTC-binding factor (CTCF) and the cohesin complex. CTCF helps define topologically associating domains (TADs) and forms chromatin insulation boundaries, ensuring the specificity of enhancer-promoter interactions and preventing aberrant gene activation [123]. In neurons, the binding of CTCF and the formation of chromatin loops are highly activity-dependent, enabling rapid and precise transcriptional responses to synaptic stimuli [78].

A growing body of evidence suggests that enhancer malfunction and disorganization of three-dimensional chromatin architecture play critical roles in the pathogenesis of neurodegenerative diseases. In disorders such as Alzheimer’s disease and Parkinson’s disease, disruptions in chromatin looping and TAD boundaries are frequently associated with downregulation of synaptic and neurotrophic genes [124,125]. The loss of enhancer-promoter connectivity may exacerbate local epigenetic alterations and contribute to widespread transcriptional dysregulation, ultimately leading to progressive neuronal dysfunction [124,125].

Importantly, the regulation of enhancer dynamics and three-dimensional chromatin structure is tightly interconnected with classical epigenetic mechanisms. Histone modifications and ATP-dependent chromatin remodeling complexes control the accessibility and activity of enhancer regions, while DNA methylation and histone methylation influence CTCF binding stability and the integrity of chromatin boundaries [126,127].

Overall, integrating enhancer dynamics and chromatin architecture into the study of neurodegenerative diseases may provide a more comprehensive understanding of the origins of transcriptional dysregulation and offer a strong theoretical foundation for identifying novel therapeutic targets and intervention strategies.

## 4. Epigenetic dysregulation as an upstream mechanistic framework in neurodegenerative pathology

The hallmark pathological features of neurodegenerative diseases include protein misfolding and aggregation, mitochondrial dysfunction and energy metabolism dysregulation, synaptic impairment, and chronic neuroinflammation [128]. Although these processes have traditionally been viewed as distinct and independent pathological pathways, emerging evidence suggests that epigenetic dysregulation may not only be a downstream consequence of neurodegeneration but also serve as a critical upstream mechanism. It potentially links genetic susceptibility, aging, and environmental stressors, driving the initiation and exacerbation of disease-related alterations across multiple levels [77].

Specifically, aberrant DNA methylation and histone modifications can alter chromatin accessibility and gene expression patterns, thereby influencing the production and clearance of aggregation-prone proteins and accelerating pathological protein deposition [129]. At the same time, imbalances in epigenetic regulation may disrupt the functional coordination between nuclear gene expression and mitochondrial activity, compromising the neuronal capacity to manage oxidative stress and meet energy demands, thus contributing to the early onset of mitochondrial dysfunction [130].

With regard to synaptic function, the accumulation of repressive chromatin marks and enhanced histone deacetylation can lead to the sustained silencing of genes involved in synaptic plasticity, weakening inter-neuronal connectivity and impairing the efficiency of information transmission [131]. In glial cells, long-term epigenetic alterations may predispose them to maintain a hyper-reactive state, transforming the inflammatory response from a transient, stress-induced repair mechanism into a chronically activated and maladaptive process [132].

Through these interconnected pathways, epigenetic mechanisms establish a more causally meaningful bridge between genetic risk, environmental influences, and neurobiological changes. This integrative framework enables the previously fragmented and independently described pathological processes to be systematically linked and understood within a unified conceptual model.

### 4.1. Alzheimer’s disease: Aβ–Tau axis–driven epigenetic reprogramming

Within the conceptual framework described above, Alzheimer’s disease (AD)exhibits a series of epigenetic alterations with adaptive features, primarily involving key pathways related to Aβ clearance, Tau pathology progression, and the maintenance of synaptic function [133]. AD is pathologically characterized by extracellular deposition of amyloid beta (Aβ) plaques and intracellular accumulation of abnormally aggregated Tau protein. An increasing body of evidence indicates that these pathological features do not arise in isolation, but instead occur within a specific epigenetic context and display a degree of disease specificity [133].

Multiple epigenome-wide association studies have identified widespread and stable abnormalities in DNA methylation patterns in the brains of AD patients. These epigenetic changes are not only correlated with pathological burden but are also closely associated with cognitive decline [134,135]. In Tau-related mechanisms, in addition to well-established abnormalities in post-translational modifications, several regulatory factors involved in Tau phosphorylation and aggregation also exhibit distinct methylation changes. For example, aberrant DNA methylation has been observed in the MAPT gene and its upstream regulatory pathways across multiple brain regions. Hypermethylation of the DUSP22 promoter region leads to transcriptional repression and is considered a key factor amplifying Tau-mediated neurotoxicity [136,137].

Parallel to Tau-related pathways, the capacity for Aβ clearance is also strongly influenced by epigenetic regulation. Numerous studies have shown that the expression or enzymatic activity of neprilysin (NEP) is reduced in aging and in the brains of AD patients, a change that is closely associated with abnormal accumulation of Aβ [138]. Epigenetic mechanisms are believed to participate in the transcriptional regulation of NEP. *In vitro* studies have suggested that Aβ exposure can induce increased DNA methylation at the NEP promoter region, accompanied by downregulation of NEP expression [139]. However, findings regarding NEP promoter methylation in human AD brain tissue remain inconsistent, and its precise role in AD pathogenesis requires further clarification [138].

Cognitive deterioration in AD is closely linked to impaired synaptic plasticity. In this context, increased DNA methylation at the promoter of brain-derived neurotrophic factor (BDNF), together with abnormal upregulation of histone deacetylase 2 (HDAC2), leads to sustained repression of genes essential for learning and memory [140,141]. This persistent chromatin repression has been recognized as a critical mechanism underlying synaptic dysfunction in neurons [141].

Against this background, the role of polyphenolic compounds in AD is more likely to involve targeted modulation of these specific pathological pathways rather than nonspecific, broad-spectrum neuroprotection. For instance, polyphenols may enhance Aβ clearance by promoting NEP expression or alleviate synaptic gene silencing by inhibiting HDAC2 activity, thereby exerting epigenetic effects at key regulatory nodes [141]. Notably, a growing number of bulk and single-cell multiomics studies have revealed pronounced cell type and brain region specificity in epigenomic remodeling within the AD brain. Changes in chromatin accessibility and associated epigenetic modifications do not occur uniformly across all cell types, but are instead concentrated in specific vulnerable neuronal subpopulations and in glial cells in activated or exhausted states. These alterations are accompanied by a progressive decline in epigenomic stability and information content as the disease advances [142].

Such dynamic epigenetic changes may help explain interindividual differences in cognitive performance among patients with similar pathological burdens. They also suggest that epigenetic interventions targeting key molecules such as NEP, BDNF, and HDAC2 may achieve more pronounced therapeutic effects within selectively vulnerable cell populations [142].

### 4.2. Parkinson’s disease: coordinated imbalance between α-synuclein derepression and repressive chromatin programs

Within the same epigenetic framework, Parkinson’s disease (PD) exhibits a pattern of dysregulation distinct from that of AD. The epigenetic abnormalities in PD are primarily centered on disrupted regulation of alpha-synuclein expression and the persistent activation of repressive chromatin states. PD is pathologically characterized by progressive degeneration of dopaminergic neurons in the substantia nigra and the formation of Lewy bodies, in which increased expression and aggregation of alpha-synuclein encoded by the SNCA gene are widely recognized as key pathogenic factors [137].

Unlike the epigenetic alterations observed in AD, which are dominated by impaired amyloid beta clearance and synaptic gene silencing, susceptibility in PD is more prominently reflected in two interrelated directions. These include dysregulated DNA methylation within SNCA regulatory regions and enhanced histone H3 lysine 9 dimethylation mediated by EHMT1 and EHMT2[143, 144]. Together, these abnormalities drive a cascade of increased SNCA expression, synaptic dysfunction, and heightened neurotoxicity [143,144]. Numerous studies have shown that the SNCA promoter and intron 1 regions in PD patients frequently exhibit persistent hypomethylation. This epigenetic state is closely associated with elevated alpha-synuclein expression and variability in clinical manifestations, and it has been observed in both sporadic and certain familial forms of PD. Such hypomethylation is therefore considered a recurrent and characteristic epigenetic alteration in PD [145]. Mechanistically, altered DNA methylation within the SNCA locus affects the binding of methylation-sensitive reader proteins, thereby influencing the recruitment and activity of transcriptional complexes. As a result, aberrant DNA methylation is directly translated into dysregulated alpha-synuclein expression [146].

In addition to DNA methylation changes, abnormal accumulation of alpha-synuclein can further enhance the activity of EHMT1 and EHMT2, leading to enrichment of H3K9me2 marks at promoters of genes involved in synaptic function. This modification further represses their transcription and converts proteotoxic stress into sustained chromatin-level suppression, representing a key mechanism underlying synaptic impairment [53]. Evidence from animal models supports the modifiability of this pathway, demonstrating that the EHMT1 and EHMT2 mediated H3K9me2 program is not merely a marker of pathology but an active driver of disease progression [53].

Taken together, epigenetic imbalance in PD is mainly characterized by disordered regulation of SNCA expression and abnormal reinforcement of chromatin repression mechanisms. This overall pattern differs markedly from the epigenetic features of AD, which are typified by reduced NEP expression and increased HDAC2 activity.

Within this context, the potential role of polyphenolic compounds is more likely to lie in modulating SNCA expression levels and maintaining relative chromatin equilibrium, rather than directly targeting a single pathogenic pathway [147,148]. In other words, polyphenols may exert regulatory effects that buffer expression imbalance and chromatin abnormalities, thereby influencing disease progression in a modulatory rather than determinative manner [147]. Furthermore, single-cell multiomics studies have revealed that elevated SNCA expression and altered accessibility of its regulatory elements are concentrated within specific vulnerable neuronal subtypes, providing direct evidence for how gene regulatory abnormalities lead to cell type specific functional imbalance [149]. In addition, animal studies have demonstrated that targeted epigenetic interventions can reverse abnormal SNCA expression, indicating that this pathway possesses genuine reparative potential [150].

Therefore, future research should integrate epigenetic, transcriptional, and genetic background information from a cell type specific perspective. Such efforts should also be combined with peripheral, clinically accessible biomarkers such as proteins and metabolites in cerebrospinal fluid or plasma. By leveraging multiomics integration and machine learning approaches, it will be possible to more systematically advance PD subtyping, biomarker discovery, and the analysis of interindividual variability in nutritional interventions such as polyphenol-based strategies.

## 5. The multi-target neuroprotective potential of polyphenols *via* epigenetic mechanisms

**Figure 2.**
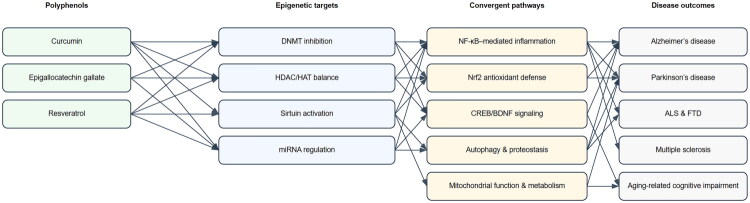
Integrative schematic of epigenetic mechanisms underlying polyphenol-mediated neuroprotection.

Polyphenolic compounds are a class of naturally occurring small molecules widely present in plant-based foods, with major dietary sources including fruits, vegetables, tea, spices, and legumes [184]. A growing body of research indicates that the neuroprotective effects of these compounds extend far beyond their traditional roles as antioxidants or anti-inflammatory agents [11]. Instead, they exert profound influences on neuronal function and cell fate through multilayered and reversible epigenetic regulatory mechanisms [11,171,172,174]. Figure 2 provides an integrative schematic of the epigenetic mechanisms involved in polyphenol-induced neuroprotection.

As outlined in previous sections, DNA methylation, histone modifications, and non-coding RNAs constitute the core epigenetic mechanisms involved in the regulation of gene expression in the central nervous system. Building upon this foundation, accumulating evidence consistently demonstrates that different types of dietary polyphenols exert neuroprotective effects primarily through a convergent set of epigenetic pathways [185–188]. These mechanisms typically include inhibition of DNA methyltransferase (DNMT) activity, thereby alleviating aberrant hypermethylation at the promoter regions of neurotrophic and antioxidant-related genes [171]. In parallel, polyphenols help restore the dynamic balance between histone acetyltransferases (HATs) and histone deacetylases (HDACs), improving chromatin accessibility and promoting reactivation of silenced genes [172,173]. Additionally, polyphenols frequently act through key signaling hubs such as SIRT1–NF-κB, collectively suppressing chronic neuroinflammation and finely tuning neuroprotective signaling pathways *via* the remodeling of non-coding RNA expression profiles, including microRNAs [173,189,190]. Table 1 summarizes a comparative analysis of representative polyphenols with respect to their epigenetic targets, key pathways, and disease relevance.

**Table 1.** Comparative and integrative analysis of epigenetic regulatory mechanisms of representative polyphenols in neurodegenerative diseases.

Polyphenol	Core epigenetic targets	Convergent pathway mechanisms	Compound-Specific advantages	Major neurodegenerative diseases	Representative references
Curcumin	DNMTs ↓; HDAC1/3/8 ↕; HATs (p300/CBP) ↓; SIRT1 ↑; miR-146a ↑	Suppression of NF-κB–mediated inflammatory responses; activation of Nrf2-dependent antioxidant pathways; enhancement of CREB/BDNF signaling; regulation of autophagy	Context-dependent bidirectional regulation of HATs/HDACs; pronounced promotion of microglial M1-to-M2 polarization; prominent roles in neurogenesis and pain-related chromatin remodeling	AD, PD, stroke, neuropathic pain, Fragile X syndrome	[151–155]
Epigallocatechin-3-gallate (EGCG)	HDAC1/2 ↓; DNMT1 ↓ (pathology-dependent); stress-related miRNAs (miR-200b, miR-125b, miR-9)	Activation of antioxidant gene transcription; NEP-mediated Aβ clearance; inhibition of NF-κB-driven neuroinflammation	Direct binding to and stabilization of misfolded proteins (TDP-43, α-synuclein, Aβ), thereby inhibiting aggregation; pronounced immunomodulatory effects (Th17/Treg balance)	AD, PD, ALS, FTD, MS	[147,156–161]
Resveratrol	SIRT1 ↑; DNMT1/3a/3b ↓; HDACs ↓; miR-132/124 ↑; miR-34a/155 ↓	Activation of the AMPK–SIRT1 axis; suppression of NF-κB signaling; improvement of mitochondrial function and energy metabolism	Tight coupling between epigenetic regulation and energy metabolism; modulation of cell-cycle–related chromatin states *via* the TET2–CDKN2A axis	AD, PD, aging-related cognitive impairment	[162–170]
Shared epigenetic regulatory framework mediated by polyphenols	DNMT inhibition; dynamic balance of HDACs/HATs; activation of sirtuins; remodeling of miRNA regulation	↓ chronic neuroinflammation; ↑ antioxidant defense; ↑ neurotrophic support; ↑ maintenance of protein homeostasis	Polyphenols act as regulators characterized by multi-target and cooperative epigenetic modulation rather than single-target agents	AD, PD, ALS, MS, etc.	[11,171–179]
Cross-disease integration of epigenetically regulated pathways in neurodegeneration	Epigenetic dysregulation of inflammation-, oxidative stress-, and synaptic function–related genes	NF-κB, Nrf2, CREB/BDNF, autophagy-related pathways	Pathway preferences of individual polyphenols determine their selective efficacy under distinct disease contexts	Multiple neurodegenerative diseases	[180–183]

*Notes.*

↓ indicates inhibition or downregulation; ↑ indicates activation or upregulation; ↕ indicates bidirectional or context-dependent regulation.

DNMTs: DNA methyltransferases; HDACs: histone deacetylases; HATs: histone acetyltransferases; NEP: neprilysin; AD: Alzheimer’s disease; PD: Parkinson’s disease; ALS: amyotrophic lateral sclerosis; MS: multiple sclerosis; FTD: frontotemporal dementia.

It is important to note that although representative polyphenols such as curcumin, epigallocatechin-3-gallate (EGCG), and resveratrol share overlapping epigenetic targets, they differ significantly in terms of regulatory intensity, pathway specificity, and molecular selectivity. For example, they exhibit distinct capacities in DNMT inhibition, subtype-selective modulation of HDACs, reactivation of genes involved in amyloid clearance, and SIRT1-dependent regulation, each demonstrating unique mechanistic features [173,185–188].

Therefore, the following sections will focus on examining the distinct epigenetic regulatory profiles and key experimental findings associated with these representative polyphenols in models of neurodegenerative disease. This will provide a clearer understanding of their individual neuroprotective advantages and potential applications in future therapeutic strategies.

### 5.1. Neuroprotective effects of curcumin in neurodegenerative diseases

#### 5.1.1. Chemical properties and neuroprotective potential of curcumin

**Figure 3.**
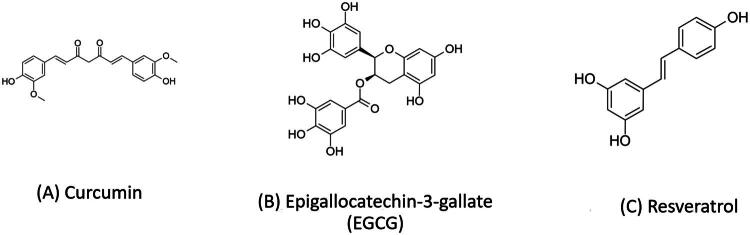
Chemical structures of representative polyphenols discussed in this review. (A) Curcumin; (B) Epigallocatechin-3-gallate (EGCG); (C) Resveratrol. Chemical structures were drawn based on data from ChemSpider (CSID: 839564, 58575, and 392875).

Curcumin is a representative polyphenolic compound derived from the rhizome of turmeric. Its unique chemical structure consists of two aromatic phenolic rings linked by an α,β-unsaturated diketone moiety, forming a highly conjugated electronic system with lipophilic properties [191]. The chemical structure of curcumin is shown in Figure 3A.

#### 5.1.2. Antioxidant activity and defense against oxidative stress

In neurodegenerative diseases, excessive accumulation of reactive oxygen species (ROS) can lead to mitochondrial dysfunction, enhanced lipid peroxidation, and DNA damage, thereby triggering inflammatory responses and cell apoptosis [192]. Curcumin has been shown to effectively alleviate oxidative stress-related tissue damage by increasing superoxide dismutase (SOD) activity, elevating levels of reduced glutathione (GSH), and enhancing total antioxidant capacity (TAC)[193]. In addition, curcumin can regulate the methylation status of the Nrf2 promoter and the acetylation levels of histones, thereby enhancing the transcriptional activity of antioxidant genes. Through these epigenetic mechanisms, curcumin helps maintain redox homeostasis at the molecular level [151,194].

#### 5.1.3. Anti-inflammatory effects and regulation of neuroinflammation

At the same time, curcumin suppresses inflammation-related signaling pathways mediated by Toll-like receptors (TLRs), including NF-κB and mitogen-activated protein kinase (MAPK) pathways, thereby reducing the production of proinflammatory cytokines such as interleukin 1 beta (IL-1β), tumor necrosis factor alpha (TNF-α), and interleukin 6 (IL-6), and preventing excessive microglial activation [195,196]. In microglial cells, curcumin inhibits the TLR4/MyD88/NF-κB signaling pathway and promotes the phenotypic transition from the M1 proinflammatory state to the M2 anti-inflammatory state, thus modulating the development of neuroinflammation and protecting neurons from sustained injury [197].

#### 5.1.4. Regulation of protein misfolding and autophagy

In the regulation of protein misfolding, curcumin can form noncovalent complexes with Aβ and alpha-synuclein, thereby inhibiting the formation of toxic oligomers and preventing mitochondrial damage and endoplasmic reticulum stress [198]. In addition, curcumin induces the expression of heat shock factor 1 (HSF-1), which upregulates molecular chaperone proteins such as heat shock protein 70 (HSP70), enhancing the cellular capacity to recognize, refold, and clear misfolded proteins [199].

In addition, curcumin can modulate cellular autophagy under specific pathological conditions [200]. Studies have shown that in HT-22 cells treated with amyloid beta 1–42 (Aβ1–42), curcumin slightly downregulates the expression of the autophagy-related factor Beclin-1 and reduces the formation of autophagosomes. This suppression of excessive autophagy may help alleviate Aβ-induced cytotoxicity, thereby improving cell viability and mitigating neuronal injury and degeneration [200].

#### 5.1.5. Neurogenesis and neural regeneration potential

In the regulation of neurogenesis and neural stem cell fate, curcumin activates classical signaling pathways such as Wnt/β-catenin and cAMP response element-binding protein/brain-derived neurotrophic factor (CREB/BDNF), thereby promoting the proliferation and directed differentiation of neural stem cells and stimulating regenerative potential under conditions such as stroke [201–204]. In addition, curcumin enhances the expression of neurogenesis-related genes, including neurogenin 1 (Neurog1), by increasing chromatin accessibility, thereby facilitating the restoration of neural function [205,206].

#### 5.1.6. Epigenetic regulatory mechanisms of curcumin

On the epigenetic level, curcumin participates in the neuroprotective process through multiple mechanisms. Curcumin can decrease CpG island methylation within the promoter areas of crucial genes by modulating upstream regulatory elements such as specificity protein 1 (Sp1) [152,207].

In AD model cells, curcumin effectively reduces the hypermethylation level of the neprilysin (NEP) gene promoter, thereby restoring its expression [152]. As an enzyme closely associated with the degradation of Aβ, the upregulation of NEP not only enhances the cellular capacity for Aβ clearance but also inhibits the phosphorylation of protein kinase B (AKT), subsequently blocking the activation of the downstream NF-κB pathway. This suppression leads to reduced expression of inflammatory mediators such as cyclooxygenase-2 (COX-2) and inducible nitric oxide synthase (iNOS), collectively alleviating the intracellular inflammatory state [152].

In terms of histone modifications, curcumin exhibits bidirectional regulatory effects on chromatin structure. Acting as a natural inhibitor of HATs, curcumin suppresses the activity of HAT family members such as p300 and CREB-binding protein (CBP), thereby reducing acetylation levels at key histone sites including H3K9ac and H4K5ac [154,208–210]. This resulting hypoacetylated state promotes chromatin condensation and subsequently represses the transcriptional activation of inflammation-related genes such as cyclooxygenase-2 (COX-2) and BDNF [154].

On the other hand, curcumin can inhibit certain HDACs, including HDAC1, HDAC3, and HDAC8, leading to increased acetylation levels of histones H3 and H4, which facilitates chromatin relaxation. At the same time, curcumin is also capable of suppressing HATs such as p300 and CREB-binding protein (CBP) while promoting the restoration of HDAC2 under specific conditions, thereby exhibiting an environment-dependent bidirectional regulatory effect [151].

In neuropathic pain models, this regulatory mechanism is particularly pronounced. Chronic nerve injury induces the enrichment of p300/CBP at promoter regions of pain-related genes, enhancing H3K9 acetylation (H3K9ac) and thereby promoting the transcription of sensitization-associated genes [154]. Curcumin has been shown to inhibit the binding of p300 to target gene promoters and reduce H3K9ac levels, effectively alleviating mechanical and thermal hyperalgesia [154].

During neural development and neural stem cell differentiation, curcumin modulates the balance between HAT (histone acetyltransferase) and HDAC (histone deacetylase) activities, thereby influencing histone acetylation status [211]. This regulation promotes the differentiation of neural progenitor cells toward a neuronal lineage, while reducing their tendency to differentiate into glial cells [211].

By dynamically regulating histone acetylation states, curcumin can exert both protective and inhibitory effects depending on the cellular context [151]. Within the nervous system, it demonstrates strong target specificity and functional diversity, highlighting its significant neuroprotective and therapeutic potential [151–153,212].

#### 5.1.7. SIRT1- and miRNA-mediated fine regulation

Curcumin has been shown to upregulate the expression and activity of the NAD^+^-dependent deacetylase SIRT1, which in turn regulates energy metabolism, oxidative stress tolerance, and inflammatory responses through the deacetylation of transcription factors such as FOXO3a, p53, and NF-κB [151–154,208,213]. This SIRT1-associated regulatory pathway contributes to the establishment of a stable neuroprotective state during chronic disease progression.

At the post-transcriptional level, curcumin exerts fine-tuned regulation over key neural pathways by modulating microRNA (miRNA) expression. Specifically, curcumin upregulates miR-146a, leading to suppression of TRAF6 and IRAK1 expression and subsequent negative regulation of the NF-κB signaling pathway. In parallel, curcumin acts synergistically with miR-101 to inhibit the expression and translation of amyloid precursor protein (APP), thereby reducing Aβ42 production [200,201,209]. The coordinated interplay among miRNAs, DNA methylation, and histone modifications collectively constitutes a multilayered and reversible epigenetic regulatory network [156].

Overall, curcumin exerts coordinated antioxidant, anti-inflammatory, and epigenetic regulatory effects in the CNS by scavenging ROS, activating Nrf2, inhibiting TLR4/NF-κB/MAPK signaling pathways, and modulating DNA methylation, histone modifications, and microRNA (miRNA) expression. Through these mechanisms, curcumin promotes neural plasticity and repair.

#### 5.1.8. Critical appraisal and limitations

Although curcumin has demonstrated notable neuroprotective potential across various models of neurodegenerative diseases, its underlying mechanisms of action remain complex and insufficiently predictable. On one hand, curcumin exhibits pronounced bidirectional epigenetic modulation, which is highly dependent on cell type and microenvironmental context. This is especially evident in its regulation of histone acetylation, where its effects on histone acetyltransferases (HATs), such as p300/CBP, and histone deacetylases (HDACs) often vary across experimental systems, thereby increasing the complexity of mechanistic interpretation [151,154,208–210].

On the other hand, although curcumin’s regulation of DNA methylation at specific gene loci (e.g. the NEP promoter) has been shown to be functionally significant, there remains a lack of comprehensive genome-wide assessments. As a result, its overall capacity to remodel the epigenomic landscape is still uncertain [152]. Related neuroprotective effects and their associated epigenetic regulatory mechanisms are systematically summarized in Table 2.

**Table 2.** Neuroprotective and epigenetic mechanisms of curcumin in neurodegenerative diseases.

Mechanistic category	Key molecular targets/signaling pathways	Regulatory mechanisms	Major functional outcomes	References
Antioxidant defense	Nrf2, SOD, GSH, TAC	Promotes Nrf2 promoter demethylation and histone acetylation; enhances antioxidant enzyme activity	Increases antioxidant capacity, reduces ROS, lipid peroxidation, and DNA damage	[151,192–194]
Anti-inflammatory regulation	TLR4/MyD88/NF-κB, MAPK	Inhibits NF-κB and MAPK signaling; modulates microglial M1/M2 polarization	Reduces pro-inflammatory cytokine production and alleviates neuroinflammation	[195–197]
DNA methylation modulation	NEP, Sp1	Decreases CpG methylation in the NEP promoter; upregulates NEP expression	Enhances Aβ clearance and suppresses inflammation-related gene expression	[152,207]
Histone modification regulation	p300, CBP, HDACs	Inhibits HAT activity and modulates HDAC levels, altering H3/H4 acetylation states	Regulates transcription of inflammatory and neurotrophic genes, restoring neuronal plasticity	[151,154,208–211]
microRNA regulation	miR-146a, miR-101	Upregulates anti-inflammatory miRNAs; suppresses TRAF6, IRAK1, and APP expression	Inhibits NF-κB–mediated inflammation and reduces Aβ generation	[213,215–217]
SIRT1-mediated signaling	SIRT1, FOXO3a, NF-κB, p53	Activates NAD⁺-dependent deacetylation and modulates stress-response transcription factors	Enhances neuronal homeostasis, anti-apoptotic capacity, and stress resistance	[218–221]
Neurogenesis and repair	Wnt/β-catenin, CREB/BDNF, Notch	Increases chromatin accessibility and activates neural stem cell differentiation pathways	Promotes neuroregeneration, synaptic formation, and functional recovery	[153,155,201–206,211]

Nrf2: Nuclear factor erythroid 2–related factor 2; SOD: Superoxide dismutase; GSH: Glutathione; TAC: Total antioxidant capacity; TLR4: Toll-like receptor 4; MyD88: Myeloid differentiation primary response protein 88; NF-κB: Nuclear factor kappa-light-chain-enhancer of activated B cells; MAPK: Mitogen-activated protein kinase; NEP: Neprilysin; Sp1: Specificity protein 1; p300, E1A-associated protein p300; CBP: CREB-binding protein; HDACs: Histone deacetylases; miRNA, microRNA; TRAF6: TNF receptor–associated factor 6; IRAK1: Interleukin-1 receptor–associated kinase 1; APP: Amyloid precursor protein; SIRT1, Sirtuin 1; FOXO3a: Forkhead box O3a; p53: Tumor protein p53; Wnt: Wingless/Integrated signaling pathway; β-catenin: Beta-catenin; CREB: cAMP response element-binding protein; BDNF: Brain-derived neurotrophic factor; Notch, Notch signaling pathway; Aβ: Amyloid-beta peptide; ROS: Reactive oxygen species.

Moreover, curcumin participates in the regulation of neuroinflammation and proteostasis through modulation of microRNAs such as miR-146a and miR-101. However, these miRNAs target broad gene networks, and their regulatory effects are highly influenced by factors such as developmental stage, pathological status, and brain region specificity, introducing substantial spatiotemporal variability [156,200,201,209]. While some studies suggest that curcumin may exert multifaceted effects, including the induction of autophagy and the promotion of neuroregeneration, many of these findings are derived from short-term or acute intervention models. Whether these effects can be sustained or are functionally relevant during chronic and progressive neurodegenerative processes remains to be fully validated [204].

More importantly, curcumin faces significant pharmacokinetic limitations, including poor bioavailability, rapid metabolism, and limited central nervous system exposure. In addition, variations across studies in terms of dosage, administration routes, and duration of intervention further compromise the comparability and consistency of results [214].

Taken together, future research must leverage human-derived cellular models, long-term intervention studies, and high-throughput epigenetic profiling technologies to better elucidate the context-dependent and target-specific characteristics of curcumin’s actions. Such efforts will be critical to establishing a more robust evidence base for its clinical translation.

### 5.2. Neuroprotective effects of epigallocatechin-3-gallate (EGCG)

#### 5.2.1. Overview of neuroprotective effects

Epigallocatechin-3-gallate (EGCG), the principal bioactive polyphenolic compound in green tea, exerts neuroprotective effects through multiple pathways that modulate key pathological processes in neurodegenerative diseases such as AD, PD, and amyotrophic lateral sclerosis (ALS) [222–224]. Overall, EGCG has been shown to delay disease progression, improve cognitive and motor functions, promote neuronal survival, and alleviate neuroinflammation [222–224]. The molecular structure of epigallocatechin-3-gallate (EGCG) is illustrated in Figure 3B.

#### 5.2.2. Anti-inflammatory regulation

In the regulation of neuroinflammation, EGCG exerts systemic inhibitory effects on multiple proinflammatory signaling pathways, particularly NF-κB、MAPK、TXNIP-NLRP3 pathways [156,222]. Through these mechanisms, EGCG reduces the expression of inflammatory cytokines such as interleukin-1 beta (IL-1β), tumor necrosis factor alpha (TNF-α), and interleukin-6 (IL-6), while preventing the polarization of microglia from the homeostatic M2 phenotype to the proinflammatory M1 phenotype, thereby mitigating the neurotoxic inflammatory microenvironment [156,222].

In BV2 microglial cells, EGCG significantly suppresses inflammation induced by lipopolysaccharide (LPS) and Aβ. This effect is achieved by inhibiting the generation of ROS, restoring mitochondrial membrane potential, and blocking TXNIP-mediated activation of the NLRP3 inflammasome, which together attenuate the progression of neuroinflammation [156]. Furthermore, EGCG upregulates protein kinase D1 (PKD1) while inhibiting Parthanatos by reducing poly(ADP-ribose) polymerase 1 (PARP-1) and apoptosis-inducing factor (AIF) levels, thereby alleviating oxidative stress and inflammation in the substantia nigra, promoting dopaminergic neuronal survival, and improving motor dysfunction in PD rat models [157].

#### 5.2.3. Regulation of mitochondrial function and autophagy

Mitochondrial dysfunction represents a critical pathological feature of neurodegenerative diseases, and EGCG has demonstrated reparative effects on multiple fronts. EGCG enhances ATP synthesis efficiency, reduces mitochondrial reactive oxygen species (mtROS) production, and promotes mitochondrial biogenesis by activating key regulators such as nuclear respiratory factor 1 (NRF1) and mitochondrial transcription factor A (TFAM), thereby improving cellular metabolic resilience [225]. In addition, EGCG activates autophagic pathways, contributing to the maintenance of protein homeostasis and mitigating oxidative stress–induced mitochondrial damage [159].

#### 5.2.4. Immunomodulation and attenuation of neuroinflammation

In rat models of PD, EGCG protects dopaminergic neurons in the substantia nigra and alleviates motor dysfunction by upregulating the protein kinase D1 (PKD1) signaling pathway. This regulation inhibits the occurrence of parthanatos and reduces oxidative stress and inflammatory responses, thereby exerting significant neuroprotective effects [157].

*In vitro* experiments, EGCG binds to the RNA recognition motif of TDP-43 and stabilizes its structure, significantly delaying the aggregation process and reducing the formation of amyloid aggregates. This finding provides molecular-level evidence supporting the potential therapeutic application of EGCG in neurodegenerative diseases such as ALS and frontotemporal dementia (FTD) [158].

In models of MS, EGCG modulates CD4^+^ T cell subsets by suppressing Th1 and Th17 cells while enhancing regulatory T (Treg) cell activity. This immune modulation attenuates myelin-specific inflammatory responses, reduces inflammatory infiltration and demyelination in the CNS, and ultimately alleviates neuronal and axonal damage, leading to improvement in disease symptoms [159].

#### 5.2.5. Regulation of protein misfolding and aggregation

In the regulation of protein misfolding and aggregation, EGCG exhibits a unique structural intervention capability, particularly in modulating the aggregation of key pathogenic proteins such as TDP-43, α-syn, and Aβ [158]. In models of ALS and frontotemporal dementia (FTD), EGCG directly binds to the RNA recognition motif (RRM) of TDP-43, stabilizing the conformation of the RRM1 domain, delaying its nucleation process, and preventing its conversion into amyloid aggregates, thereby inhibiting pathological aggregation. This interaction does not occupy the RNA-binding site but instead stabilizes the protein conformation through aromatic stacking and hydrogen bonding, forming non-aggregating complexes that reflect EGCG’s ability to modulate protein structural dynamics [158].

Molecular dynamics simulations and nuclear magnetic resonance (NMR) chemical shift perturbation experiments further confirm the interaction between EGCG and the flexible linker region as well as key aromatic residues of TDP-43, revealing the diversity and stability of this binding mode [158]. Similar mechanisms are observed in the regulation of α-syn and Aβ aggregation, where EGCG promotes the conversion of toxic oligomers into non-toxic, amorphous aggregates and disrupts beta-sheet formation, thereby protecting neurons from oligomer-induced cytotoxic stress [159].

#### 5.2.6. Epigenetic regulatory mechanisms

In terms of epigenetic regulation, the neuroprotective effects of epigallocatechin-3-gallate (EGCG) are primarily reflected at the levels of histone modification and non-coding RNA regulation. Compared to its more clearly defined DNA demethylation effects observed in cancer models, the direct impact of EGCG on DNA methylation in the central nervous system remains controversial. Its functional significance may depend on specific cell types and pathological contexts [158].

In contrast, more definitive evidence indicates that EGCG exerts neuroprotective effects through the regulation of histone modifications. The key mechanism involves the inhibition of histone deacetylase (HDAC) activity or expression, leading to elevated histone acetylation levels, chromatin relaxation, and transcriptional activation of neuroprotective genes [160]. In AD models, EGCG exhibits properties similar to those of HDAC inhibitors, particularly showing pronounced inhibition of HDAC1. This inhibition results in a relaxed chromatin conformation in the promoter region of the neprilysin (NEP) gene, enhancing the recruitment of transcription factors and transcriptional complexes, thereby upregulating NEP mRNA and protein expression, promoting Aβ clearance, and improving cognitive function [160].

Under oxidative or toxic stress conditions, EGCG downregulates DNMT1 and HDAC2 while upregulating stress-responsive miRNAs such as miR-200b [147]. These changes promote histone acetylation and transcriptional activity at the promoters of antioxidant genes, accompanied by global DNA hypomethylation, thus creating an epigenetic environment favorable for neuroprotection [147]. Furthermore, studies suggest that EGCG-mediated inhibition of HDAC2 is closely associated with increased histone acetylation at the promoters of antioxidant-related genes, such as superoxide dismutase 2 (SOD2) and glutathione peroxidase 1 (GPX1), thereby enhancing cellular antioxidant defenses [147].

In addition to DNA methylation and histone modification, the regulation of non-coding RNAs by EGCG represents an important component of its epigenetic effects. Multiple studies have shown that EGCG can reshape miRNA expression profiles, thereby modulating key pathological processes such as Aβ deposition, oxidative stress, and inflammatory responses at the post-transcriptional level [226]. Among these, miR-125b has emerged as a central regulatory node, displaying pronounced model- and context-dependent effects. In SH-SY5Y cells and APP/PS1 transgenic mice, EGCG treatment downregulates miR-125b expression, which is accompanied by cognitive improvement. Conversely, in Neuro2a cells and in the serum of certain transgenic mouse models, an upregulation of miR-125b has been observed, suggesting that EGCG exerts direction-dependent modulation of this miRNA, potentially influenced by cell type, disease stage, and stress conditions [226].

Meanwhile, studies have also found that EGCG exerts a restorative effect on the regulation of molecules such as miR-9 and miR-191–5p in the serum of APP/PS1 mice, further highlighting its systemic impact on circulating miRNA levels [227].

This type of epigenetic modification affects not only gene expression within neurons but also the fate determination of immune cells. For example, it can enhance the expression of the transcription factor forkhead box P3 (Foxp3), which is associated with regulatory T (Treg) cells, thereby increasing immune tolerance and attenuating the spread of central inflammation in multiple sclerosis (MS) [159,161].

Overall, as a natural polyphenol, EGCG can influence the initiation and development of neurodegenerative disorders through multiple mechanisms, including epigenetic regulation and modulation of signaling pathways. Its comprehensive regulatory capacity provides strong support for future precision interventions, disease course attenuation, and functional improvement.

Table 2: Neuroprotective and Epigenetic Mechanisms of Epigallocatechin-3-Gallate (EGCG) in Neurodegenerative Diseases

#### 5.2.7. Critical appraisal and limitations

As a natural epigenetic modulator with multi-target activity, epigallocatechin-3-gallate (EGCG) has attracted considerable attention for its neuroprotective potential in neurodegenerative diseases. However, current studies exhibit substantial divergence in terms of result consistency and reproducibility. On one hand, most mechanistic investigations of EGCG rely heavily on BV2 microglial cells, transgenic animal models, or acute injury paradigms. Marked differences in model construction, stress conditions, and treatment protocols across studies have led to pronounced variability in findings related to key regulatory processes such as miRNA modulation and DNA methylation [156,157,159]. For example, contradictory effects of EGCG on the expression of miR-125b and miR-132 have been reported, suggesting that its regulatory outcomes may be highly dependent on specific cellular metabolic states, pathological environments, or experimental conditions [147,226,227]. Related neuroprotective effects and their associated epigenetic regulatory mechanisms are systematically summarized in Table 3.

**Table 3.** Neuroprotective and epigenetic mechanisms of epigallocatechin-3-gallate (EGCG) in neurodegenerative diseases.

Mechanistic category	Key molecular targets/signaling pathways	Regulatory mechanisms	Major functional outcomes	References
Anti-inflammatory regulation	NF-κB, MAPK, TXNIP-NLRP3	Suppresses pro-inflammatory signaling; blocks TXNIP-mediated NLRP3 inflammasome activation; limits M1 microglial polarization	Reduces IL-1β, TNF-α, IL-6 expression; alleviates neuroinflammation	[156,157,222]
Oxidative-stress modulation	ROS, PKD1, PARP-1/AIF	Inhibits ROS generation and parthanatos; restores mitochondrial membrane potential	Protects dopaminergic neurons, improves motor function (PD models)	[156,157]
Protein misfolding regulation	TDP-43, α-synuclein, Aβ	Binds to RNA-recognition motif of TDP-43; stabilizes conformation; converts toxic oligomers to non-toxic amorphous aggregates	Prevents amyloid and α-syn aggregation; protects neurons from proteotoxicity	[158,159]
DNA-methylation modulation	DNMT1, COMT, SAM/SAH cycle	Inhibits DNMT1 directly and indirectly *via* COMT-mediated SAM depletion	Potential reactivation of silenced promoters; role in CNS remains under investigation	[228]
Histone modification regulation	HDAC1, HDAC2	Inhibits HDAC activity/expression; increases histone acetylation at neuroprotective gene promoters (e.g. NEP, SOD2, GPX1)	Promotes NEP expression, enhances antioxidant gene transcription, improves cognition	[147,160]
microRNA regulation	miR-200b, miR-125b, miR-9, miR-191–5p	Alters miRNA profiles in a context-dependent manner	Modulates Aβ deposition, oxidative stress, and inflammatory signaling	[147,226,227]
Immune modulation (MS models)	Th1/Th17 cells, Treg cells, Foxp3	Suppresses Th1/Th17 differentiation; enhances Treg activity	Reduces demyelination and CNS inflammation; improves clinical symptoms	[159,161]
Mitochondrial regulation & autophagy	NRF1, TFAM, mtROS	Activates mitochondrial biogenesis and autophagic pathways	Enhances ATP production and metabolic resilience; maintains protein homeostasis	[159,225]

NF-κB: Nuclear factor kappa-light-chain-enhancer of activated B cells; MAPK: Mitogen-activated protein kinase; TXNIP: Thioredoxin-interacting protein; NLRP3: NOD-like receptor family pyrin domain-containing 3; IL-1β: Interleukin-1 beta; TNF-α: Tumor necrosis factor alpha; IL-6: Interleukin-6; ROS: Reactive oxygen species; PKD1: Protein kinase D1; PARP-1: Poly(ADP-ribose) polymerase 1; AIF: Apoptosis-inducing factor; TDP-43: TAR DNA-binding protein 43; α-syn: Alpha-synuclein; Aβ: Amyloid-beta peptide; DNMT1: DNA methyltransferase 1; COMT: Catechol-O-methyltransferase; SAM: S-adenosylmethionine; SAH: S-adenosylhomocysteine; HDAC1: Histone deacetylase 1; HDAC2: Histone deacetylase 2; NEP: Neprilysin; SOD2: Superoxide dismutase 2; GPX1: Glutathione peroxidase 1; miR: microRNA; Th1: T helper type 1 cell; Th17: T helper type 17 cell; Treg: Regulatory T cell; Foxp3: Forkhead box P3; NRF1: Nuclear respiratory factor 1; TFAM: Mitochondrial transcription factor A; mtROS: Mitochondrial reactive oxygen species; ATP: Adenosine triphosphate; CNS: Central nervous system; PD: Parkinson’s disease.

Furthermore, although EGCG is proposed to participate in DNA methylation regulation through DNMT1 inhibition or by altering the SAM/SAH ratio, its epigenetic mechanisms, while theoretically plausible, lack systematic validation regarding stability and disease specificity within the nervous system [147,158]. The potential of EGCG to interfere with pathological protein aggregation is largely attributed to its non-covalent interactions with misfolded proteins such as alpha-synuclein, amyloid beta, and TDP-43. However, most of these observations originate from *in vitro* molecular-level studies, and whether such interactions exhibit sufficient specificity and sustained efficacy within the complex intracellular environment remains to be conclusively demonstrated [158,159].

From a pharmacokinetic perspective, EGCG is limited by poor oral absorption, rapid metabolism, and restricted central nervous system exposure, which significantly constrain its translational potential. In addition, substantial heterogeneity across studies in terms of dosage, intervention duration, and experimental animal strains poses further challenges for cross-study comparison and mechanistic synthesis [156,157,159].

Therefore, future research should prioritize quantitative investigations based on dose–response relationships, combined with standardized experimental designs and multicenter validation, to enhance the scientific rigor and clinical translational value of EGCG-related studies [156,157,159].

### 5.3. Neuroprotective effects of resveratrol in Neurodegener5.3.1 overview of the neuroprotective effects of resveratrol (RSV)

Resveratrol (RSV), a natural polyphenolic compound, has been increasingly recognized for its neuroprotective effects that are closely linked to epigenetic mechanisms [229]. In particular, during neural aging and the progression of neurodegenerative diseases such as AD and PD, epigenetic imbalance is considered a key factor driving pathological development [230,231]. The actions of RSV extend beyond its traditional antioxidant and anti-inflammatory properties, as it can reshape neuronal functional states at the molecular level through multilayered and dynamic epigenetic mechanisms [232]. In this way, RSV exerts systemic neuroprotective effects and helps delay disease progression. The chemical structure of resveratrol is presented in Figure 3C.

#### 5.3.2. Antioxidant and anti-inflammatory mechanisms

RSV exhibits remarkable neuroprotective effects in the nervous system. It has been reported that it not only suppresses oxidative stress and inflammatory responses and enhances cell survival but also improves neuronal function by modulating mitochondrial activity and dynamics [162]. In AD models, RSV activates AMPK and inhibits mTOR, thereby promoting the clearance of abnormal proteins such as Aβ. At the same time, through SIRT1-dependent regulation, RSV improves transcriptional and metabolic responses, helping to maintain neuronal survival and cognitive function [162].

#### 5.3.3. Representative epigenetic mechanism

The most well-characterized epigenetic feature of resveratrol (RSV) is its ability to activate the NAD^+^-dependent deacetylase SIRT1. By enhancing SIRT1 activity, RSV regulates the acetylation status of various transcription factors and chromatin-associated proteins, thereby reshaping neuronal stress responses and inflammatory states at the transcriptional level [164].

At the level of DNA methylation, RSV inhibits the activity of DNMTs, including DNMT1, DNMT3a, and DNMT3b, thereby significantly reducing the abnormal hypermethylation of promoters of neuroprotective genes such as estrogen receptor alpha (ERα) and restoring their normal expression [163,233]. This process is directly linked to neuronal synaptic plasticity, trophic support, and survival capacity.

At the same time, RSV plays a crucial role in regulating histone modifications. RSV markedly increases active histone marks such as H3K9ac and H3K27ac, enhancing the transcriptional accessibility of anti-inflammatory genes and neurotrophic factors [163]. This action reverses chromatin repression caused by chronic inflammation or aging, thereby maintaining neuronal functional stability [163].

#### 5.3.4. Epigenetic regulation of the cell cycle and aging

Resveratrol (RSV) also exhibits distinct advantages in regulating neuronal cell cycle and aging-related pathways. Studies have shown that RSV can inhibit the expression and activity of TET2, thereby increasing DNA methylation at the CDKN2A promoter region and reducing the expression of the cell cycle inhibitor p16^INK4a [165]. This regulation alleviates the inhibition of CDK4, promotes the phosphorylation and functional restoration of retinoblastoma protein (pRb), and ultimately reduces aberrant neuronal cell cycle re-entry and susceptibility to apoptosis [165]. This mechanism provides important epigenetic evidence for the neuroprotective effects of RSV in the context of neuronal aging and neurodegenerative processes.

In inflammatory conditions, resveratrol (RSV) suppresses the transcriptional activity of the NF-κB p65 (RelA) subunit through SIRT1-mediated deacetylation, leading to a significant reduction in the expression of pro-inflammatory cytokines such as IL-1β, TNF-α, and IL-6. This, in turn, inhibits microglial overactivation and alleviates the sustained stimulation of the NLRP3 inflammasome [166,167,229].

#### 5.3.5. Post-Transcriptional regulation mediated by miRNAs

At the post-transcriptional level, resveratrol (RSV) further refines its epigenetic regulatory network by remodeling microRNA (miRNA) expression profiles. RSV upregulates miR-132 and miR-124, which are closely associated with neuronal plasticity and dendritic spine formation, thereby enhancing synaptic connectivity and cognitive function [168,234]. Concurrently, it downregulates pro-inflammatory or pro-apoptotic miRNAs such as miR-34a and miR-155, thereby relieving their suppressive effects on SIRT1, antioxidant pathways, and neurotrophic factors [169,170]. This miRNA–SIRT1 co-regulatory mechanism contributes to the maintenance of neuronal functional homeostasis under chronic pathological conditions.

Overall, resveratrol (RSV) exerts its neuroprotective effects in neurodegenerative diseases through a SIRT1-centered epigenetic regulatory axis, which coordinately modulates DNA methylation, histone modifications, inflammatory signaling, and miRNA networks. This multi-target, reversible, and highly adaptable mode of action highlights RSV’s potential in both the prevention and treatment of neurodegenerative disorders, offering promising avenues for future therapeutic development.

#### 5.3.6. Critical appraisal and limitations

The therapeutic potential of resveratrol (RSV) in neurodegenerative disease intervention has been widely recognized, particularly for its ability to activate the SIRT1 signaling axis involved in chromatin remodeling and inflammation regulation [164]. However, this mechanism-dependent feature serves as both a key strength and a significant limitation [162,229–231]. On one hand, SIRT1, as a metabolism-sensitive deacetylase, exhibits considerable variability in expression levels and functional status across different brain regions, cell types, and stages of disease progression. As a result, the biological effects of RSV are characterized by substantial individual variability and context dependence [162,229–231]. Related neuroprotective effects and their associated epigenetic regulatory mechanisms are systematically summarized in Table 4.

**Table 4.** Neuroprotective and epigenetic mechanisms of resveratrol (RSV) in neurodegenerative diseases.

Mechanistic category	Key molecular targets/signaling pathways	Regulatory mechanisms	Major functional outcomes	References
Antioxidant and metabolic regulation	AMPK, mTOR, SIRT1	Activates AMPK and inhibits mTOR to promote Aβ clearance; modulates transcriptional and metabolic responses *via* SIRT1	Reduces oxidative stress and protein accumulation; maintains neuronal survival and cognitive function	[162]
DNA methylation modulation	DNMT1, DNMT3a, DNMT3b, ERα	Inhibits DNMT activity and decreases hypermethylation of neuroprotective gene promoters such as ERα	Restores neurotrophic gene expression and enhances synaptic plasticity and neuronal viability	[163,233]
Histone modification regulation	HDACs, HATs, H3K9ac, H3K27ac	Downregulates HDACs or modulates HAT activity; increases active histone marks	Relieves chromatin repression; promotes transcription of anti-inflammatory and neurotrophic genes	[163]
SIRT1-mediated signaling regulation	SIRT1, NF-κB, TET2, CDKN2A (p16^INK4a)	Deacetylates NF-κB (RelA) to suppress inflammatory signaling; inhibits TET2, enhancing CDKN2A methylation and reducing p16^INK4a expression	Decreases proinflammatory cytokines; alleviates neuronal cell-cycle arrest and apoptosis	[162,164–167,229]
microRNA regulation	miR-132, miR-124, miR-34a, miR-155	Upregulates neuroplasticity-related miRNAs (miR-132, miR-124); downregulates proinflammatory and proapoptotic miRNAs (miR-34a, miR-155)	Enhances synaptic connectivity and cognitive performance; reduces inflammation and apoptosis	[168–170,234]
Anti-inflammatory and immune modulation	NF-κB, NLRP3, IL-1β, TNF-α, IL-6	Suppresses NF-κB–mediated transcription *via* SIRT1-dependent deacetylation of RelA	Reduces microglial overactivation and NLRP3 inflammasome activity; attenuates chronic neuroinflammation	[164,166,167,229]

AMPK: AMP-activated protein kinase; mTOR: Mammalian target of rapamycin; SIRT1: Sirtuin 1; Aβ: Amyloid-beta peptide; DNMT1: DNA methyltransferase 1; DNMT3a: DNA methyltransferase 3 alpha; DNMT3b: DNA methyltransferase 3 beta; ERα: Estrogen receptor alpha; HDACs: Histone deacetylases; HATs: Histone acetyltransferases; H3K9ac: Acetylation of histone H3 at lysine 9; H3K27ac: Acetylation of histone H3 at lysine 27; NF-κB: Nuclear factor kappa-light-chain-enhancer of activated B cells; TET2: Ten-eleven translocation methylcytosine dioxygenase 2; CDKN2A: Cyclin-dependent kinase inhibitor 2 A; p16^INK4a: Inhibitor of cyclin-dependent kinase 4a; RelA: v-rel avian reticuloendotheliosis viral oncogene homolog A (NF-κB p65 subunit); miR: microRNA; NLRP3: NOD-like receptor family pyrin domain-containing 3; IL-1β: Interleukin-1 beta; TNF-α: Tumor necrosis factor alpha; IL-6: Interleukin-6.

On the other hand, although several studies have reported RSV’s influence on DNA methylation, the TET2 pathway, and key factors such as p16^INK4a, most of this evidence is confined to candidate genes or specific signaling nodes. There remains a lack of systematic, genome-wide, and temporally resolved analyses based on large sample sizes, limiting our understanding of its broader epigenetic impact [163,165,233].

At the level of non-coding RNAs, RSV modulates multiple miRNA networks to finely regulate neuroinflammation and synaptic plasticity. However, these miRNA pathways are often involved in a wide array of physiological and pathological processes, making the regulatory network highly complex. Potential off-target effects and directional reversals of regulation at different disease stages introduce further uncertainty in mechanistic interpretation [168–170,234].

From a translational medicine perspective, RSV also faces several pharmacokinetic limitations, including poor water solubility, low bioavailability, and rapid metabolism, which severely restrict its effective exposure in the central nervous system [229,232]. Although advanced delivery strategies such as liposomal encapsulation and prodrug design have been proposed in recent years, most of these efforts remain at the proof-of-concept stage. Comprehensive toxicological evaluations and validation in large animal models are still lacking.

Moreover, substantial variation across studies in terms of formulation selection, administration routes, and intervention duration further complicates the long-term assessment of RSV’s safety and efficacy.

In summary, to advance the clinical translation of RSV in neurodegenerative diseases, it is essential not only to deepen our understanding of the boundaries of SIRT1-dependent regulation, but also to pursue more systematic and refined research in areas such as delivery technology optimization, population heterogeneity analysis, and the design of long-term follow-up studies.

## 6. Discussion and future perspectives

### 6.1. Clinical evidence from randomized controlled trials and meta-analyses

In recent years, multiple randomized controlled trials (RCTs) and meta-analyses have systematically evaluated the potential effects of curcumin, resveratrol, and green tea polyphenols in neurodegenerative diseases such as Alzheimer’s disease (AD) and Parkinson’s disease (PD). Most of these studies have focused on cognitive function, memory performance, and disease progression. However, overall findings generally report only mild or statistically insignificant improvements.

For example, in clinical research on curcumin, a double-blind, randomized, placebo-controlled preliminary trial involving 34 AD patients used the Mini-Mental State Examination (MMSE) as the primary endpoint over a 6-month intervention period, while also monitoring plasma isoprostane, serum Aβ40, and antioxidant levels. The results showed no significant differences between curcumin and placebo groups in terms of MMSE scores or biochemical markers, with only partial elevation in certain antioxidant indices, but no clear cognitive benefits [235].

Another randomized, double-blind trial conducted by Ringman et al. involving 36 patients with mild to moderate AD used the Alzheimer’s Disease Assessment Scale–Cognitive Subscale (ADAS-Cog) as the primary outcome. After 24 weeks of follow-up, no significant improvements were observed in ADAS-Cog, MMSE, Neuropsychiatric Inventory (NPI), or Alzheimer’s Disease Cooperative Study–Activities of Daily Living (ADCS-ADL). Additionally, there were no notable changes in plasma or cerebrospinal fluid levels of Aβ, tau, or oxidative stress markers [236].

Systematic reviews and meta-analyses have further confirmed this trend. For instance, one analysis including six RCTs with a total of 289 participants found a statistically significant decline in MMSE scores in the curcumin group (SMD = −0.90), with no positive signals in key biomarkers or disease progression [237]. Another meta-analysis of eight RCTs involving 389 participants similarly found no clear effect on overall cognition or AD subgroups, with only minor improvements in secondary cognitive domains such as working memory, but a higher incidence of gastrointestinal adverse reactions [238].

Moreover, a systematic review by Wang et al. that included nine RCTs showed a slight overall positive trend in effect size. However, due to high heterogeneity, no statistically significant benefits were observed in AD patients or studies using MMSE or ADAS-Cog as outcome measures. These findings suggest that the observed improvements may primarily arise from small sample sizes, formulations with enhanced bioavailability, or specific subgroups, making it difficult to draw definitive conclusions [239].

In studies on resveratrol, a phase II multicenter, randomized, double-blind, placebo-controlled trial by Turner et al. (119 patients with mild to moderate AD, 52-week follow-up) reported certain differences in some biomarkers and neuroimaging outcomes. However, there were no significant changes in MMSE, ADAS-Cog, Clinical Dementia Rating–Sum of Boxes (CDR-SB), ADCS-ADL, or NPI. Only slight and unstable improvements were observed in daily living activities, and the observed brain volume reductions on imaging were not associated with clinical improvements [240]. Related systematic reviews have also indicated that resveratrol has limited cognitive impact across different populations, with most core cognitive measures showing no significant differences, and only minor benefits noted in secondary outcomes such as delayed recognition [241].

Regarding green tea polyphenols, the PROMESA study evaluated the potential disease-modifying effects of epigallocatechin-3-gallate (EGCG) in patients with multiple system atrophy (MSA) (*n* = 92, 52-week follow-up). Results showed no superiority of the EGCG group over placebo in primary endpoints including Unified Multiple System Atrophy Rating Scale (UMSARS) motor score, total UMSARS score, and Clinical Global Impression (CGI). Exploratory MRI analyses revealed a possible reduction in the rate of atrophy in specific brain regions, but the sample size was very small, and no corresponding clinical improvements were observed. Additionally, the EGCG group had a higher incidence of elevated liver enzymes, highlighting the need for cautious safety evaluation when using high-dose formulations [242].

### 6.2. Translational barriers

#### 6.2.1. Bioavailability, pharmacokinetics, and BBB constraints

From a translational medicine perspective, the largely negative or limited efficacy observed in the aforementioned clinical studies may be influenced by multiple factors. First, polyphenolic compounds generally exhibit low oral bioavailability and rapid metabolism *in vivo*. Different polyphenols also show considerable variation in absorption efficiency, plasma half-life, and formulation dependence. Insufficient systemic exposure has been identified as one of the key factors limiting their clinical effectiveness [243].

For instance, studies have shown that curcumin has extremely low oral bioavailability due to its high hydrophobicity, poor intestinal absorption, and significant first-pass metabolism. Free curcumin is almost undetectable in plasma, and its elimination half-life is short. Although novel formulations such as nanoemulsions and liposomes can significantly enhance its relative bioavailability, systemic exposure under conventional oral preparations remains very limited [214]. Similarly, resveratrol, despite having relatively high absorption, undergoes rapid glucuronidation and sulfation in the enterohepatic circulation, resulting in very low plasma concentrations of the parent compound. Its absolute bioavailability is generally less than 1% and it is rapidly metabolized and excreted [244]. Taking EGCG as an example, its plasma exposure levels, such as AUC and Cmax, are highly influenced by dosing conditions and formulation types, indicating that its systemic availability depends heavily on intestinal absorption and the surrounding chemical environment. Overall, plasma concentrations remain low, reflecting clear limitations in its absorption and pharmacokinetics [245].

In addition, differences in physicochemical properties among various polyphenol subclasses, such as lipophilicity, molecular weight, and polarity, lead to significant differences in their ability to cross the blood-brain barrier. This further contributes to the inconsistency in clinical research outcomes [246]. Although curcumin, resveratrol, and EGCG have been reported to possess certain blood-brain barrier permeability, quantitative studies have shown that their actual concentrations in brain tissue are generally low. There is also marked heterogeneity across different polyphenol types and disease stages, making it difficult to achieve sufficient pharmacological concentrations in the central nervous system, which affects their therapeutic effects [240,247,248].

#### 6.2.2. *In vivo* exposure versus *in vitro* efficacy mismatch

Current *in vivo* studies suggest that while most polyphenols or their conjugated metabolites can cross the blood-brain barrier, their actual concentrations in the central nervous system typically remain at the nanomolar level. For example, even in Alzheimer’s disease clinical studies involving high-dose, long-term oral administration of resveratrol, the concentration of the parent compound in cerebrospinal fluid remained at a low nanomolar level, with a higher abundance of conjugated metabolites [249]. This indicates that possessing central permeability does not necessarily equate to achieving adequate pharmacological accumulation in the brain. In the case of catechins, tissue distribution studies in animal models have also shown that even with strategies such as nano-delivery, EGCG concentrations in brain tissue or cerebrospinal fluid may still be as low as approximately 1 nanomolar. For instance, reported brain levels of EGCG were about 0.6 nanograms per milliliter, which converts to around 1.3 nanomolar, suggesting that there is a significant upper limit to brain exposure [250]. These *in vivo* exposure levels are much lower than the effective concentrations typically used in *in vitro* experiments. Many cell studies employ micromolar or even higher concentrations to observe antioxidant, anti-inflammatory, or anti-aggregation effects [251]. The large gap between effective concentrations *in vitro* and the achievable concentrations *in vivo* is likely one of the key limiting factors preventing the consistent clinical translation of the neuroprotective effects of polyphenols [252].

Moreover, increasing evidence indicates that polyphenols are more commonly present in the brain in the form of conjugated metabolites, such as glucuronides and sulfates. These metabolites also tend to be present at low concentrations, usually within the nanomolar range [249]. This further complicates the interpretation of central pharmacological mechanisms based solely on the parent compounds.

#### 6.2.3. Gut microbiota–dependent metabolism and interindividual heterogeneity

Microbiota-mediated metabolic regulation is a critical yet often overlooked factor influencing the systemic exposure and central delivery of polyphenols. The biological effects of polyphenols *in vivo* largely depend on their metabolic transformation by the gut microbiota, which generates phenolic acid metabolites that may differ significantly from the parent compounds in terms of absorption efficiency and blood-brain barrier permeability [253]. After ingestion, dietary polyphenols are typically present in conjugated forms and are only partially absorbed in the small intestine [246]. A substantial portion reaches the colon, where they serve as substrates for microbial metabolism. Through microbial enzymatic activity, polyphenols are converted into structurally simpler, low-molecular-weight phenolic metabolites that are more readily absorbed into the systemic circulation [246].

Once in the bloodstream, both polyphenols and their microbial metabolites can undergo further Phase II metabolism in the liver, affecting their distribution and exposure profiles within the body [246]. Current evidence suggests that only certain gut microbiota-derived phenolic metabolites possess the capacity to cross the blood-brain barrier, and their accessibility to brain tissues is further constrained by physicochemical properties and transport mechanisms [246]. Moreover, interindividual variability in gut microbiota composition has been identified as a potential source of the high heterogeneity observed in the clinical efficacy of polyphenols [254].

Additionally, considerable differences across clinical studies in terms of dosage, formulation types, and duration of intervention contribute to further heterogeneity in outcomes and introduce uncertainty in efficacy assessment [255].

#### 6.2.4. Safety concerns and off-target effects

In terms of safety, polyphenolic compounds are not entirely devoid of risk. Some studies have indicated that under high doses or specific conditions, certain polyphenols may exhibit pro-oxidant activities and potentially interfere with drug-metabolizing enzymes or transporters, thereby posing a risk of drug-drug interactions [256]. Hepatotoxicity associated with green tea extracts and EGCG has been reported in clinical settings. For instance, elevated liver enzymes were observed in the PROMESA study, suggesting that long-term or high-dose administration may carry certain safety concerns [242,257].

Furthermore, polyphenols typically exert multi-target effects, involving a wide range of biological pathways such as antioxidant defense, anti-inflammatory activity, metabolic regulation, and mitochondrial function. The complexity of these mechanisms makes it difficult to attribute observed clinical or molecular outcomes to a single epigenetic regulatory pathway, thereby increasing the challenges and uncertainties in mechanistic interpretation [258].

### 6.3. Comparison with other epigenetic modulators

Although this review has systematically outlined how natural polyphenols modulate DNA methylation, histone modifications, and non-coding RNAs to exert epigenetic regulation in neurodegenerative diseases, from a broader perspective of epigenetic intervention strategies, their mechanisms of action differ from those of currently developed epigenetic drugs and RNA-based therapies. These differences highlight the unique advantages of polyphenols in terms of intervention pathways and therapeutic potential.

For example, epigenetic drugs such as histone deacetylase (HDAC) inhibitors typically act by directly inhibiting enzymatic activity, leading to rapid and widespread changes in chromatin accessibility. This has been shown to significantly improve phenotypes in various models of neurodegenerative disease [259]. In contrast, the regulation of histone acetylation by polyphenols is more dependent on pathological context. Their mechanisms primarily involve modulation of SIRT1 activity, regulation of the acetylation status of inflammation-related transcription factors, and influence on the availability of key metabolic cofactors such as NAD^+^ and S-adenosylmethionine (SAM). This results in a gradual restoration of aberrant epigenetic states, particularly under stress or inflammatory conditions [260,261]. Such a regulatory approach is relatively mild but more aligned with the slow, progressive nature of neurodegenerative diseases.

Similar distinctions are observed in the regulation of inflammatory transcriptional programs. The BET (bromodomain and extraterminal domain) protein family, including BRD2, BRD3, and BRD4, functions as epigenetic ‘readers’ that recognize histone acetylation marks and regulate RNA polymerase II-mediated transcription [262]. BET proteins are broadly involved in basal transcription and cellular homeostasis across various cell types. BET inhibitors, by blocking the activity of reader proteins such as BRD4, can exert strong anti-inflammatory effects with high specificity [263]. However, due to their involvement in fundamental transcriptional processes, long-term inhibition of BET proteins may pose risks to the homeostasis of neurons and glial cells [262].

In contrast, polyphenols tend to act through key regulators such as NF-κB, Nrf2, and SIRT1, which are closely linked to metabolic and redox states. By modulating multiple signaling pathways, polyphenols can lower inflammatory thresholds while exerting minimal interference with the basal transcriptional activity necessary for cellular function [264].

In terms of RNA-based therapeutics, technologies such as antisense oligonucleotides (ASOs) and siRNAs enable highly specific silencing of pathogenic genes. For example, nucleic acid-based therapies targeting tau protein have demonstrated promising target selectivity in early-stage clinical studies [265]. However, due to challenges related to delivery efficiency, treatment burden, and long-term safety, such approaches are more suitable for intermittent interventions or targeting specific pathogenic drivers [266]. By contrast, polyphenols do not rely on single-target mechanisms but act more broadly by remodeling miRNA expression profiles, mitigating inflammatory and metabolic imbalances, and restoring transcriptional and epigenetic homeostasis at a systemic level. This makes them more appropriate for early-stage intervention or long-term maintenance therapy [267].

Overall, HDAC inhibitors, BET inhibitors, and RNA-based therapies are well-suited for intensive, stage-specific interventions targeting well-defined pathological pathways. In contrast, natural polyphenols, as low-toxicity and potentially long-term epigenetic modulators, can provide homeostatic support in combination therapies, synergistically enhancing neuroprotective mechanisms and offering a more balanced profile between therapeutic efficacy and safety [268].

### 6.4. Implications for future research and clinical translation

Based on current clinical and translational evidence, future research on polyphenols in the context of neurodegenerative diseases should prioritize directions that are feasible in the short term and exhibit clear biological target specificity [269]. Rather than broadly investigating various epigenetic mechanisms, emphasis should first be placed on identifying the primary epigenetic targets of individual polyphenols. For example, studies on resveratrol could focus on its role in SIRT1-mediated deacetylation pathways, particularly the synergistic interactions between this pathway, DNA methylation, and inflammation-related transcriptional programs, in order to elucidate its critical functions in suppressing neuroinflammation and modulating neuronal aging [162,164–167,229]. EGCG offers a unique opportunity to explore its dual functionality as both a histone deacetylase (HDAC) inhibitor and a modulator of protein misfolding. Future research should dissect its mechanistic roles in regulating neprilysin (NEP) expression, facilitating Aβ clearance, and modulating the interplay between oxidative stress and inflammatory signaling [147,156–158]. In the case of curcumin, its bidirectional capacity to modulate HAT/HDAC balance and influence DNA methylation patterns suggests it may epigenetically regulate the expression of neurotrophic factors, neuroinflammatory transcriptional networks, and genes involved in neurogenesis [151–153,194].

In terms of delivery strategies, rather than broadly pursuing precision nutrition approaches, a more realistic short-term focus would be on formulation optimization and enhancing central nervous system (CNS) delivery efficiency, especially for polyphenolic compounds with an established safety profile. Employing nanocarriers and other advanced technologies to improve solubility, stability, and brain exposure, in combination with target-specific PK/PD (pharmacokinetic/pharmacodynamic) evaluation metrics, may allow for simultaneous advancement in mechanistic validation and pharmacodynamic assessment [269].

Lastly, although personalized or precision nutrition strategies hold substantial promise in the long term, their successful implementation relies heavily on integrated understanding of gut microbiota composition, genetic background, and metabolic characteristics [253–255]. At present, there remain significant challenges regarding stratification criteria and clinical feasibility, making such strategies more appropriate as mid-to-long-term research objectives, rather than immediate priorities for clinical translation [253–255].

In summary, based on current clinical evidence, it is premature to consider curcumin, resveratrol, or green tea polyphenols as clinically validated disease-modifying interventions. Their potential therapeutic value remains contingent upon improved delivery systems, enhanced systemic and CNS exposure, and more rigorous clinical validation.

## 7. Conclusion

This review systematically summarizes the multi-target neuroprotective effects of natural polyphenolic compounds in neurodegenerative diseases, with particular emphasis on their transcriptional regulatory roles mediated through epigenetic mechanisms such as DNA methylation, histone modifications, and non-coding RNAs. Studies have shown that polyphenols possess not only traditional biological activities such as antioxidant, anti-inflammatory, and mitochondrial-supporting effects, but also the ability to reshape gene expression profiles and influence cell fate in the central nervous system through reversible and dynamic epigenetic regulation.

However, current clinical studies have generally demonstrated limited intervention effects, and definitive therapeutic benefits remain difficult to establish. This is mainly due to factors such as poor oral bioavailability, limited permeability across the blood-brain barrier, insufficient effective concentrations *in vivo* compared to *in vitro* conditions, and interindividual differences in metabolism. Most trials have not observed significant cognitive improvements and have reported substantial variability and dose-dependent adverse effects, indicating that the clinical translation of polyphenolic compounds still faces considerable challenges.

In summary, natural polyphenols exhibit multidimensional neuroprotective potential through epigenetic regulation. Although translational difficulties remain, the continued advancement of epigenetic research and the integration of multidisciplinary approaches may support the development of polyphenols as a bridge between nutritional modulation and gene expression control. This provides theoretical support for individualized interventions, early-stage delay, and adjunctive treatment strategies for neurodegenerative diseases. Future research should focus on enhancing central nervous system exposure, identifying key epigenetic targets, and exploring precision nutritional strategies centered on epigenetic remodeling, thereby advancing neuroprotection research toward more systematic and controllable directions.

## Data Availability

No datasets were generated or analysed during the current study.

## Abbreviations

DNMTs: DNA methyltransferases
TETs: Ten-eleven translocation enzymes
SAM: S-adenosylmethionine
SAH: S-adenosylhomocysteine
HAT: Histone acetyltransferase
KMT: Lysine methyltransferase
HDAC: Histone deacetylase
KDM: Lysine demethylase
Ac: Acetylation
Me: Methylation
miRNA: MicroRNA
circRNA: Circular RNA
lncRNA: Long non-coding RNA
Nrf2: Nuclear factor erythroid 2–related factor 2
SOD: Superoxide dismutase
GSH: Glutathione
TAC: Total antioxidant capacity
TLR4: Toll-like receptor 4
MyD88: Myeloid differentiation primary response protein 88
NF-κB: Nuclear factor kappa-light-chain-enhancer of activated B cells
MAPK: Mitogen-activated protein kinase
NEP: Neprilysin
Sp1: Specificity protein 1
p300: E1A-associated protein p300
CBP: CREB-binding protein
TRAF6: TNF receptor–associated factor 6
IRAK1: Interleukin-1 receptor–associated kinase 1
APP: Amyloid precursor protein
SIRT1: Sirtuin 1
FOXO3a: Forkhead box O3a
p53: Tumor protein p53
Wnt: Wingless/Integrated signaling pathway
β-catenin: Beta-catenin
CREB: cAMP response element-binding protein
BDNF: Brain-derived neurotrophic factor
Notch: Notch signaling pathway
Aβ: Amyloid-beta peptide
ROS: Reactive oxygen species
TXNIP: Thioredoxin-interacting protein
NLRP3: NOD-like receptor family pyrin domain-containing 3
IL-1β: Interleukin-1 beta
TNF-α: Tumor necrosis factor alpha
IL-6: Interleukin-6
PKD1: Protein kinase D1
PARP-1: Poly(ADP-ribose) polymerase 1
AIF: Apoptosis-inducing factor
TDP-43: TAR DNA-binding protein 43
α-syn: Alpha-synuclein
DNMT1: DNA methyltransferase 1
COMT: Catechol-O-methyltransferase
HDAC1: Histone deacetylase 1
HDAC2: Histone deacetylase 2
SOD2: Superoxide dismutase 2
GPX1: Glutathione peroxidase 1
Th1: T helper type 1 cell
Th17: T helper type 17 cell
Treg: Regulatory T cell
Foxp3: Forkhead box P3
NRF1: Nuclear respiratory factor 1
TFAM: Mitochondrial transcription factor A
mtROS: Mitochondrial reactive oxygen species
ATP: Adenosine triphosphate
CNS: Central nervous system
PD: Parkinson’s disease
AMPK: AMP-activated protein kinase
mTOR: Mammalian target of rapamycin
DNMT3a: DNA methyltransferase 3 alpha
DNMT3b: DNA methyltransferase 3 beta
ERα: Estrogen receptor alpha
HATs: Histone acetyltransferases
H3K9ac: Acetylation of histone H3 at lysine 9
H3K27ac: Acetylation of histone H3 at lysine 27
TET2: Ten-eleven translocation methylcytosine dioxygenase 2
CDKN2A: Cyclin-dependent kinase inhibitor 2A
